# Gallic acid ameliorates colitis by trapping deleterious metabolite ammonia and improving gut microbiota dysbiosis

**DOI:** 10.1128/mbio.02752-23

**Published:** 2023-12-21

**Authors:** Jie Peng, Tong Liu, Pengfei Meng, Yue Luo, Siyue Zhu, Yanxin Wang, Mingxia Ma, Jiaojiao Han, Jun Zhou, Xiurong Su, Shiming Li, Chi-Tang Ho, Chenyang Lu

**Affiliations:** 1 State Key Laboratory for Managing Biotic and Chemical Threats to the Quality and Safety of Agro-products and School of Marine Science, Ningbo University, Ningbo, Zhejiang, China; 2 Department of Food Science, Rutgers University, New Brunswick, New Jersey, USA; 3 College of Biology and Agricultural Resources, Huanggang Normal University, Huangang, China,; 4 School of Food Science and Biotechnology, Zhejiang Gongshang University, Hangzhou, China; University of Texas Health Science Center, School of Public Health, Houston, Texas, USA

**Keywords:** polyphenols, gallic acid, colitis, gut microbiota, ammonia, fecal microbiota transplantation

## Abstract

Gut microbiota dysbiosis is causally related to inflammatory bowel disease (IBD), and increased levels of the gut metabolite ammonia have been proposed to contribute to IBD development. In this study, we aimed to clarify the anti-colitis mechanism of gallic acid (GA) based on its ability to trap the deleterious metabolite ammonia and improve gut microbiota. Aminated product was detected in the fecal samples of mice after oral gavage of gallic acid (GA) and identified as 4-amino-substituted gallic acid (4-NH_2_-GA), thus confirming the ability of GA to trap ammonia *in vivo*. Then, we compared the beneficial effects of GA and 4-NH_2_-GA on dextran sulfate sodium (DSS)-induced colitis mouse and found that both compounds managed to alleviate colitis phenotypes, indicating ammonia trapping had no adverse effect on the original anti-colitis activity of GA. In addition, both GA and 4-NH_2_-GA improved the gut microbiota dysbiosis induced by DSS, and fecal microbiota transplantation was subsequently performed, which further revealed that the gut microbiota mediated the anti-colitis activity of both GA and 4-NH_2_-GA. In summary, this study clarified that GA alleviated colitis by targeting both the symptoms and root causes: it directly reduced the deleterious metabolite ammonia by forming aminated metabolites without compromising the original anti-colitis activity, and it also improved gut microbiota dysbiosis, which in turn contributed to the alleviation of colitis. Since the GA structure is presented in various polyphenols as a common building block, the novel anti-colitis mechanism obtained from GA may also apply to other complex polyphenols.

**IMPORTANCE:**

The dysbiosis of the gut microbiota and its metabolism directly cause the emergence of IBD. In this study, we aimed to clarify the anti-colitis mechanism of GA in sight of gut microbiota and its metabolite ammonia. We discovered that GA directly captured and reduced the harmful metabolite ammonia in vivo to produce the aminated metabolite 4-NH2-GA, while the amination of GA had no adverse effect on its initial anti-colitis activity. In addition, both GA and its aminated metabolite improved the gut microbiota in colitis mice, and the modified gut microbiota, in turn, helped to relieve colitis. Since the GA structure is presented in diverse polyphenols as a common building block, the novel anti-colitis mechanism targeting the symptoms and root causes might also apply to other complex polyphenols.

## INTRODUCTION

Inflammatory bowel disease (IBD), a chronic and relapsing intestinal disease that poses a significant threat to the health of patients with multiple symptoms, has become a global burden in both Western societies and Asia (1). Microbiota is postulated to be a critical factor involved in the development of IBD. The absence of gut microbiota caused milder intestinal inflammation and altered epithelial injury conditions in germ-free (GF) mice with dextran sulfate sodium (DSS)-induced colitis (2). Simultaneously, a dramatic shift in the microbial structure and functions in IBD individuals was observed (3). Further studies have reported the induction of phenotypes in GF mice model after fecal microbiota transplantation (FMT) from IBD mice (4), while manipulation of microbiota via prebiotics, probiotics, and antibiotics has shown positive effects (5). Therefore, gut microbiota played a causative role in the development and alleviation of IBD. However, the underlying mechanism remained elusive.

In the last decade, researchers have proposed gut metabolites as one of the mediators involved, which is usually found to vary with changes in microbial structure. Microbial metabolites can be derived from modifying host molecules or the metabolism of dietary substrates. Common microbial metabolites, including bile acid, short-chain fatty acids (SCFAs), and tryptophan metabolites, are reported to play essential roles in immune homeostasis, energy metabolism, and mucosal integrity (3). In addition, other metabolites, such as ammonia, may also play a role in IBD. Ammonia, generally considered a waste, can be majorly produced by microbes through the hydrolysis of urea and the catabolism of amino acids. Traditionally, ammonia is known to exert neurological toxicity at high plasma levels (6). Recently, ammonia has been suggested to compromise epithelial function and possibly affect adaptive immunity due to its impact on tight junction protein barrier, phagocytosis, and immune response (7, 8). Moreover, a high-protein diet was associated with an increased likelihood of ulcerative colitis (UC) relapse and colitis severity (9), while the luminal ammonia content in high protein-fed mice was 5.5-folds higher than that in normal mice (10), thus indicating that ammonia may act as a harmful microbial metabolite during the IBD development, and reducing its level could be helpful in the maintenance of overall health.

Gallic acid (GA or 3,4,5-trihydroxybenzoic acid) is a bioactive compound that is widely distributed in fruits (grapes and berries), vegetables, and beverages (tea and wine) (11, 12), with extremely poor bioavailability (<2% of oral dose) (13). GA offers various physiological benefits (14, 15); among them, the anti-colitis effect of GA has been reported in several mouse models, either by suppressing colonic inflammation or reducing oxidative stress (16, 17). Meanwhile, GA has the characteristic structure of a *vic*-trihydroxyl group on the aromatic ring, commonly shared by many phenolic phytochemicals, such as epigallocatechin-3-gallate (EGCG) and myricetin. EGCG and myricetin can react with ammonia at the 4′ position of the *vic*-trihydroxyl group to form aminated metabolites (Fig. S1) (18, 19). Therefore, the ability of GA to react with ammonia *in vivo* is warranted, and the ability of GA to attenuate IBD by reducing ammonia and forming aminated metabolites requires further investigation. On the other hand, although gut microbiota dysbiosis was suggested to play a vital role in IBD, the effect between the gut microbiota and GA is mutual: the gut microbiota transformed GA to final bioactive derivatives, and the administration of GA modulated the composition and functions of the gut microbiota (20). However, it remained unclear whether the gut microbiota mediates the anti-colitis activity of GA and its aminated metabolite.

In this study, we proposed that reducing the harmful microbial metabolite ammonia and further fundamental improvement in gut microbiota mediated the beneficial effects of GA on colitis. Therefore, we investigated the ability of GA to trap ammonia *in vivo* as well as the structure of the potential aminated product, confirmed and compared the anti-colitis function of GA and aminated-GA [4-amino-3,5-dihydroxybenzoic acid, 4-amino-substituted gallic acid (4-NH_2_-GA)] in a mouse model, and clarified whether these beneficial effects were mediated by improving gut microbiota dysbiosis.

## RESULTS

### Aminated GA synthesis and structure identification

**Fig 1:**
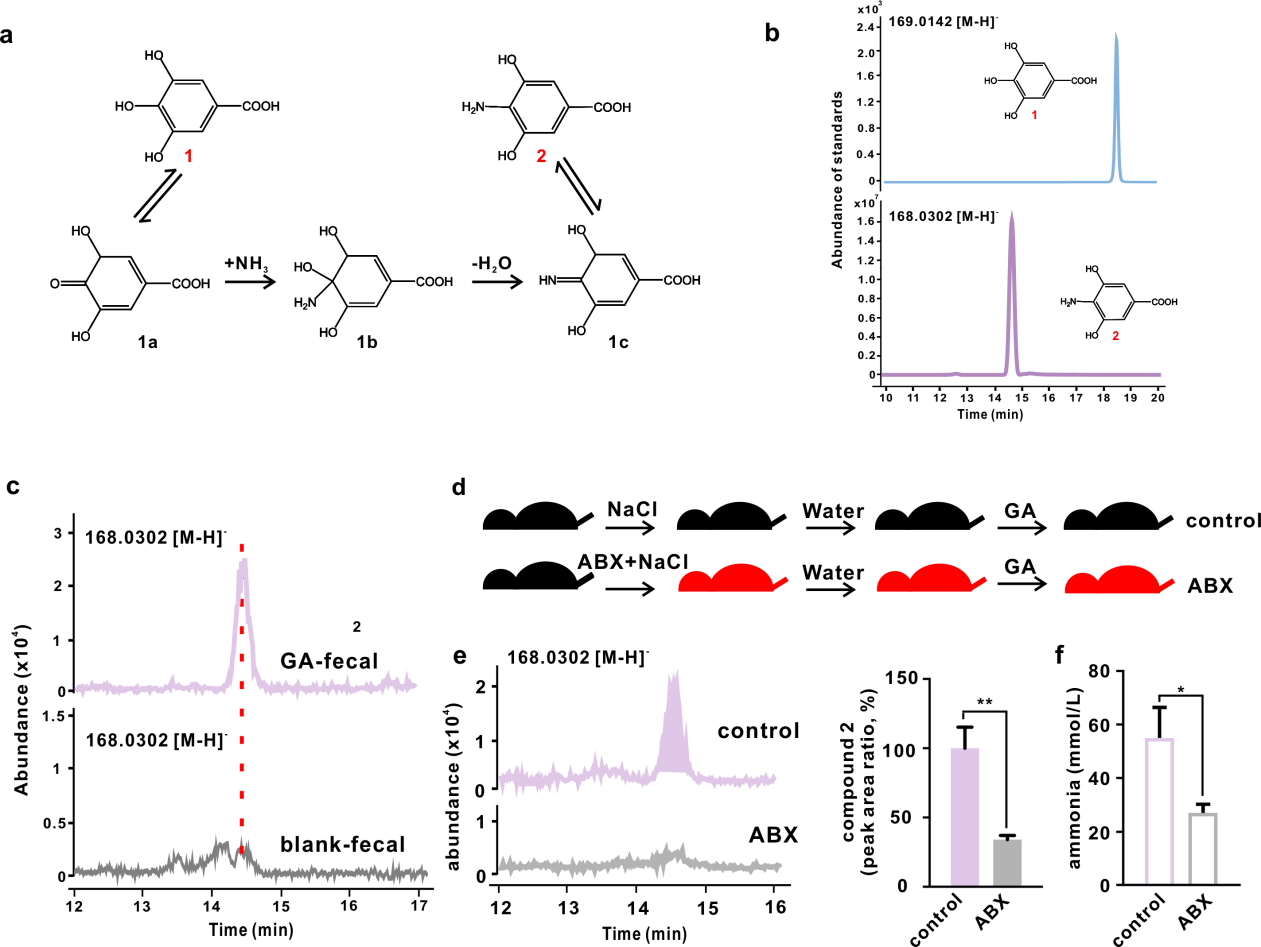
Verification of aminated GA standard and *in vivo* detection of aminated GA in both normal and antibiotic-treated mice. (a) Proposed chemical synthetic route for aminated GA. (b) Liquid chromatography-mass spectrometry (LC-MS) spectrum of standard GA (1) and aminated GA (2). (c) LC-MS spectrum of control mice feces collected after oral gavage of GA and vehicle. (d) Experimental design for antibiotic-treated mice that received GA and vehicle by gavage. (e) Detection and the normalized peak area ratio of aminated GA (2). (f) Ammonia content in the feces of control and antibiotic-treated mice. Data were represented as mean ± SD. *P < 0.05, **P < 0.01.

The aminated-GA product was synthesized by the direct reaction of GA with ammonia at room temperature in the presence of air (Fig. 1a). A new peak (retention time 14.4 min) was detected in the reaction system in the negative mode (Fig. 1b). The molecular formula was deduced as C_7_H_7_NO_4_ based on the quasi-molecular ion at *m*/*z* 168.0302 ([M-H]^−^, calcd, for C_7_H_7_NO_4_ 169.0375), which was 1 Dalton less than GA ([M-H]^−^, calcd, for C_7_H_6_O_5_ 170.0215, retention time 18.6 min). Subsequently, the structure of this aminated-GA product was identified as 4-NH_2_-GA (abbreviated as NGA in the figures) via ^1^H and ^13^C nuclear magnetic resonance (NMR) spectra (Table 1; Fig. S2), and its synthetic mechanism was proposed and depicted in Fig. 1a.

**TABLE 1:** ^1^H-(400 MHz) and ^13^C-NMR (100 MHz) data of 4-NH_2_-GA and GA

Position	4-NH_2_-GA		GA	
	*δ*_H_	*δ*_C_	*δ*_H_	*δ*_C_
	*δ* (ppm), *J* (Hz)	*δ* (ppm)	*δ* (ppm), *J* (Hz)	*δ* (ppm)
1	–	116.88	–	120.41
2	6.92 (2H, s)	108.29	6.92 (2H, s)	108.69
3	9.23 (2H, s)	143.29	9.19 (2H, s)	145.37
4	4.51 (1H, s)	129.56	8.83 (1H, s)	137.95
5	9.23 (2H, s)	143.29	9.19 (2H, s)	145.37
6	6.92 (2H, s)	108.29	6.92 (2H, s)	108.69
COOH		167.71	12.25 (1H, s)	167.41

### GA trapped ammonia to form 4-NH_2_-GA *in vivo*

To clarify whether GA can trap ammonia *in vivo* and identify potential aminated metabolites, fecal samples collected from mice receiving GA treatment were analyzed by Liquid chromatography-mass spectrometry (LC-MS) under selective ion monitoring mode. Compared to the control samples, one new peak was identified with the same profiles of retention time and tandem mass spectrum profile as the synthetic standard, while the peak area ratio between this aminated metabolite to GA is ~5% in the fecal samples (Fig. 1c; Fig. S3). Therefore, the aminated metabolite of GA was characterized as 4-NH_2_-GA, demonstrating that GA could trap ammonia *in vivo* but only in a limited portion.

### The amination of GA was mediated by gut microbiota via supplementing ammonia substrate

Previous studies have shown that gut microbiota is involved in the amination of EGCG and myricetin, yet the underlying mechanism remains elusive (18, 19). Herein, we aimed to investigate whether gut microbiota also contributes to the formation of 4-NH_2_-GA (Fig. 1d). Compared to the control mice, the relative level of 4-NH_2_-GA in antibiotics-treated mice significantly decreased by 66% (*P* < 0.01) (Fig. 1e). Meanwhile, the depletion of gut microbes resulted in a significantly reduced ammonia content in the feces of antibiotics-treated mice (*P* < 0.05) (Fig. 1f). Together, these data supported that GA trapped the ammonia and formed 4-NH_2_-GA *in vivo*, and the depletion of gut microbiota resulted in a lower level of ammonia, thus inhibiting the amination of GA.

### The amination of GA had no adverse effect on its original anti-colitis activity

**Fig 2:**
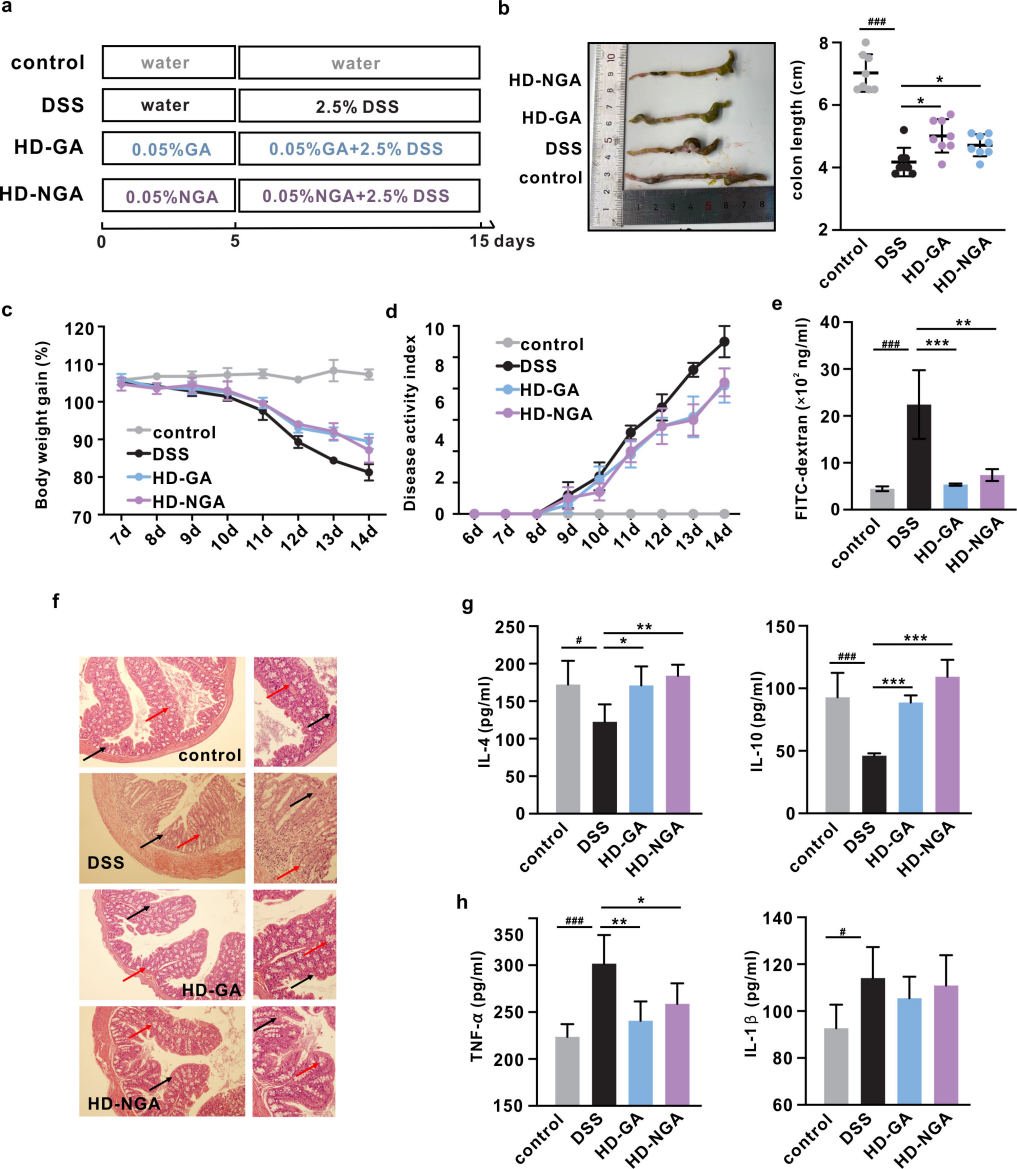
The anti-colitis activity of GA and its aminated metabolite in DSS-induced colitis mice. (a) Experimental design. (b) Representative picture and colon length. (c) Body weight gain. (d) Disease activity index. (e) Serum fluorescein Isothiocyanate-dextran content. (f) Representative image of the sectioned colon after hematoxylin and eosin staining (100× and 200×). The locations of crypts and goblet cells were marked with black and red arrows, respectively. (g) Serum level of anti-inflammatory cytokines and (h) pro-inflammatory cytokines. Data were represented as mean ± SD. ^#,^*p < 0.05, **p < 0.01, ^###,^***p < 0.001.

Next, we explored the influence of GA amination on its original anti-colitis activity in DSS-induced colitis mice (Fig. 2a; Fig. S4a). No significant differences were observed in the water or food intake between the groups (Fig. S4b and c). Compared to untreated mice, mice treated with DSS developed colitis phenotypes, which were alleviated in mice treated with GA and 4-NH_2_-GA in a dose-dependent manner, whereas no significant differences were observed between the groups treated with these two compounds at the exact dosage (Fig. 2b through 2d; Fig. S4d and e). Moreover, similar to GA, 4-NH_2_-GA exhibited the ability to significantly improve intestinal permeability and colon histological parameters in mice with colitis at the highest dosage (Fig. 2e and f; Fig. S4f). Serum levels of anti-inflammatory cytokines [interleukin (IL)-4 and IL-10] significantly increased in 4-NH_2_-GA- and GA-treated mice (Fig. 2g; Fig. S5a and b), while the levels of tumor necrosis factor alpha (TNF)-α (*P* < 0.05) and IL-1β (*P* > 0.05) decreased (Fig. 2h; Fig. S5c and d). In addition, the 4-NH_2_-GA treatment restored the protein levels of IL-4 and IL-10 in colon tissue to a level closer to that of the control group than the GA treatment (Fig. S5e). Together, these data showed that the amination of GA had no adverse effect on its original anti-colitis activity.

### The amination of GA enhanced its specific regulatory effect on signaling pathways

**Fig 3:**
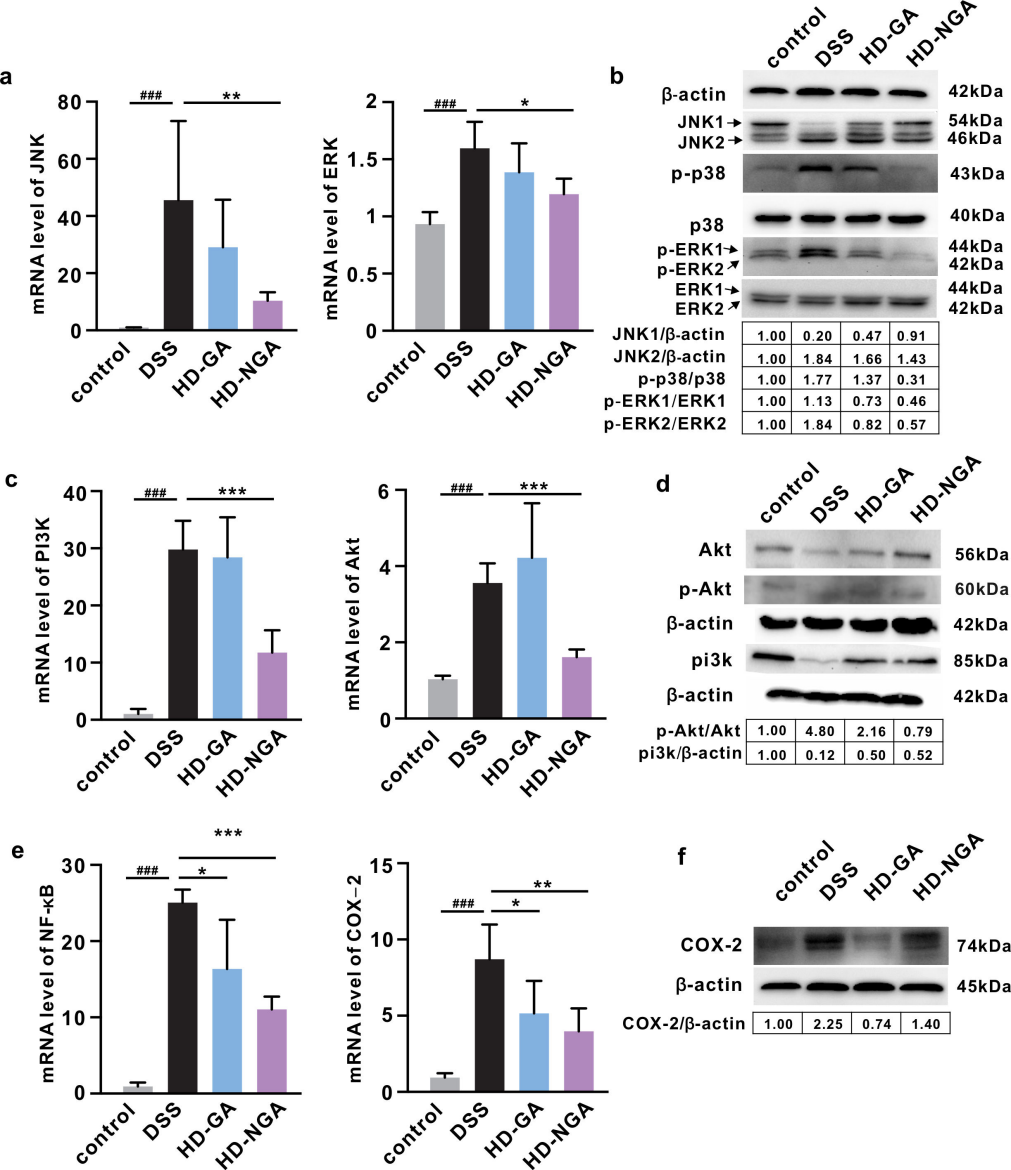
Regulatory effects of GA and its aminated metabolite on signaling pathways, demonstrated by mRNA levels of (a) JNK and ERK, (c) PI3K and Akt, and (e) NF-κB and COX-2, as well as Western blot image and further quantification of protein expression of (b) JNK, p38, p-p38, ERK, and p-ERK, (d) Akt, p-Akt, PI3K, and (f) COX-2. Protein expressions are all normalized to β-actin. Data were represented as mean ± SD. *P < 0.05, **P < 0.01, ^###,^***P < 0.001.

Significant upregulation of the nuclear factor kappa B (NF-κB), mitogen-activated protein kinases (MAPKs), and phosphoinositide 3-kinase (PI3K)/protein kinase B (Akt) signaling pathways were observed after DSS treatment in mRNA levels. The former two pathways were significantly downregulated by both 4-NH_2_-GA and GA treatments at high doses, while the last pathway was only significantly restored by 4-NH_2_-GA treatment (Fig. 3a, c and e; Fig. S5f). In addition, DSS treatment elevated protein levels of COX-2 and JNK2 and increased phosphorylation of Akt, p38, and ERK, which were almost all restored to control levels in 4-NH_2_-GA- and GA-treated mice (Fig. 3b, d, and f). Together, these data demonstrated that the amination of GA enhanced its inhibitory effects on PI3K/Akt and MAPKs signaling pathways.

### GA and its aminated metabolite improved the DSS-induced microbiota dysbiosis

**Fig 4:**
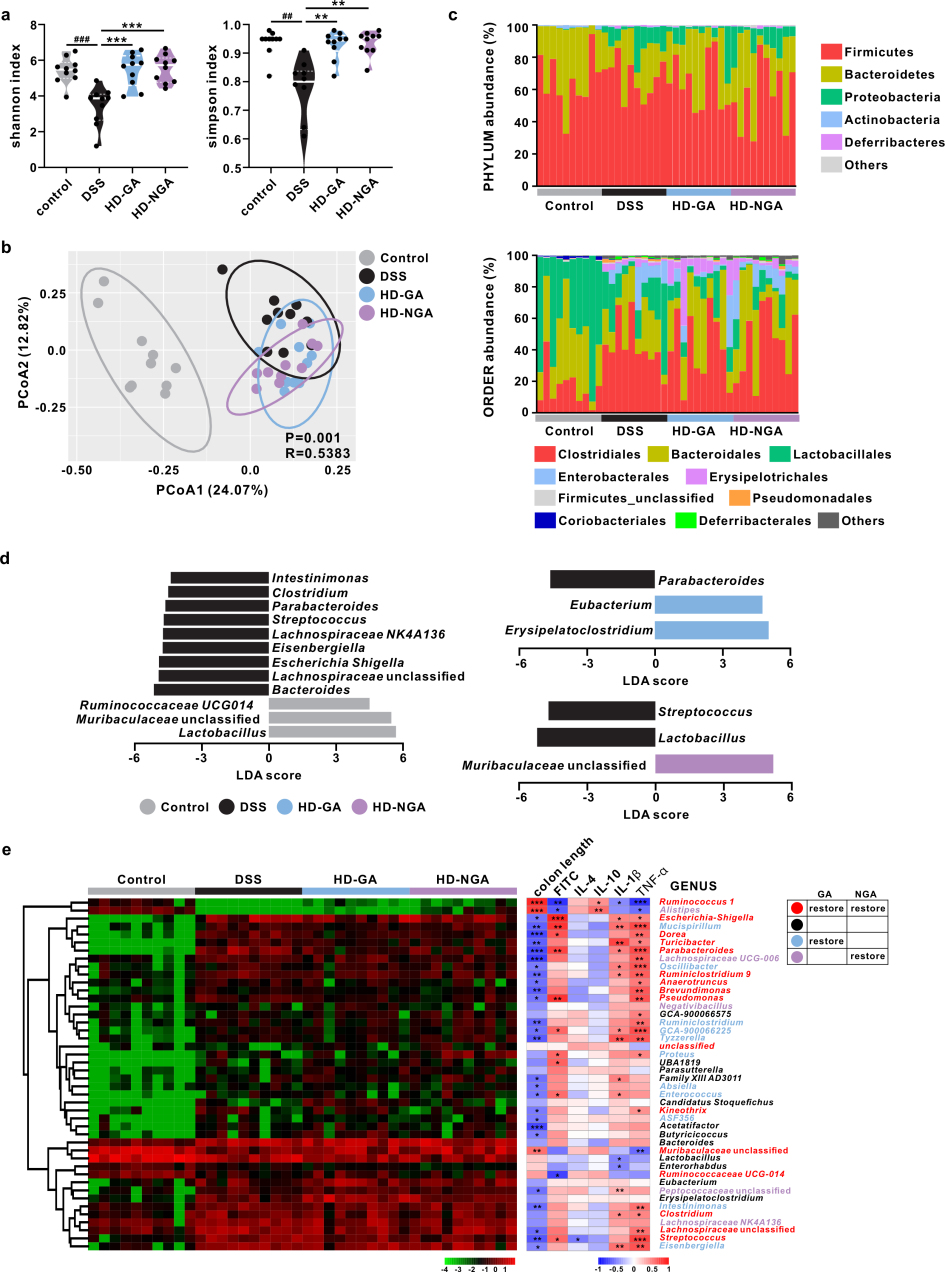
Gut microbiota modulation in compound supplementation experiment. (a) α-Diversity (Shannon and Simpson index). (b) UniFrac-based PCoA analysis. (c) Gut microbiota profile at phylum and order levels. (d) Biomarkers identified by linear discriminant analysis effect size analysis (*P* < 0.05, LDA >4 or <−4). (e) Heatmap of 44 key genera responding to different treatments and correlation study between gut microbiota and colitis-related indices. *P < 0.05, ^##,^** *P* < 0.01, ^###^***P < 0.001.

The DSS group exhibited significantly reduced α-diversity compared to the control group, whereas GA and 4-NH_2_-GA significantly increased the bacterial richness and diversity (Fig. 4a; Fig. S6a and b). In addition, principal coordinates analysis (PCoA) result showed that the gut microbiota in DSS-treated mice was separated away from that of mice in the control group, while both GA and 4-NH_2_-GA treatments trended to shift the gut microbiota pattern from one more similar to the DSS group to that of the control group, while the changes were not identical (Fig. 4b). At the phylum level, GA restored the abundance of all major phyla except Actinobacteria, while 4-NH_2_-GA treatment only restored the abundance of Bacteroidetes and Firmicutes. At the order level, GA treatment restored the abundance of Clostridiales, Enterobacterales, and Bacteroidales, whereas 4-NH_2_-GA treatment restored only Bacteroidales (Fig. 4c; Table S2). Additionally, among these differentially abundant genera identified by linear discriminant analysis effect size algorithm, the GA group was enriched in *Erysipelatoclostridium* and *Eubacterium*, whereas 4-NH_2_-GA was enriched in unclassified Muribaculaceae. Among these three genera, only unclassified Muribaculaceae was increased in the control group compared to the DSS group, while *Erysipelatoclostridium* and *Eubacterium* showed no differential abundance (Fig. 4d). Among 44 key genera identified in response to different treatments, GA and 4-NH_2_-GA treatments restored 27 and 21 genera, respectively, to the control level, and 16 genera were restored by both treatments. Spearman rank correlation analysis showed that 6 and 34 genera were negatively and positively correlated with the severity of colitis (*P* < 0.05), respectively (Fig. 4e; Table S3). These data indicated that GA and its aminated metabolite modulated the gut microbiota in DSS-treated mice.

### The anti-colitis activity of GA and its aminated metabolite is mediated by gut microbiota

**Fig 5:**
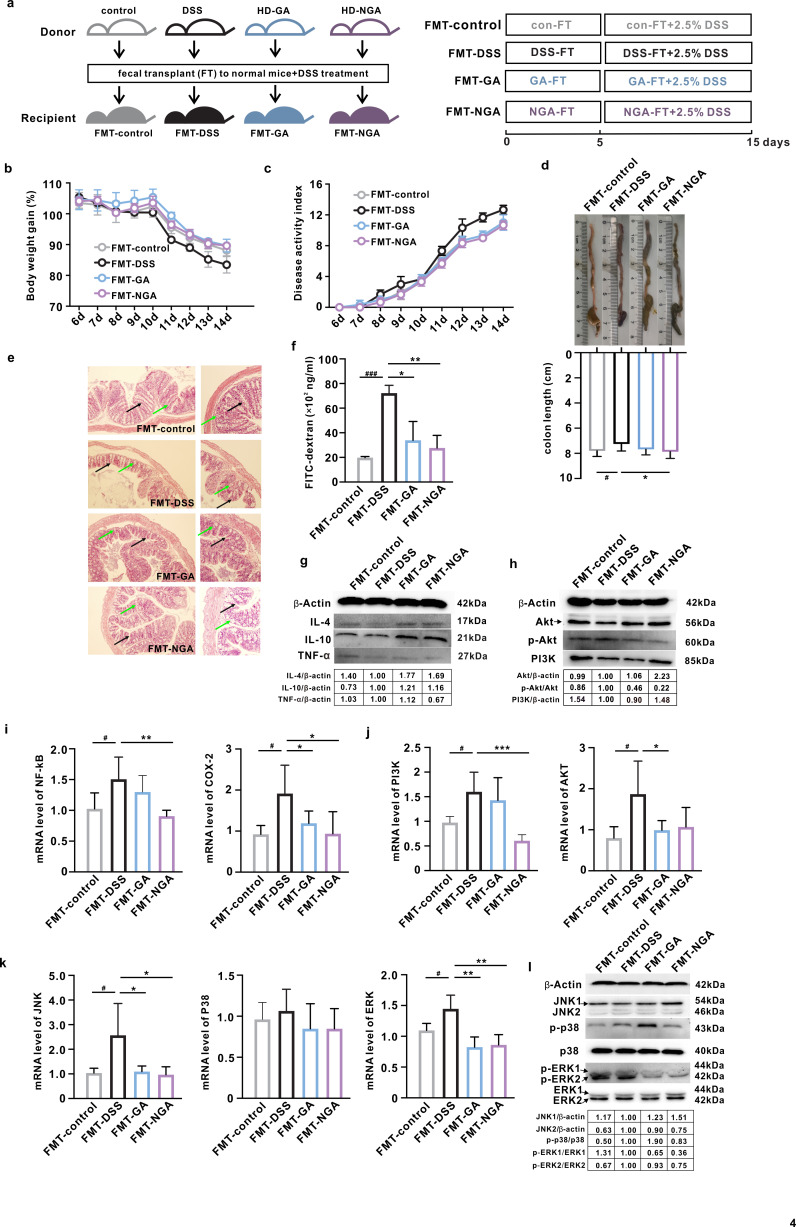
The anti-colitis activity of FMT from corresponding donors treated with GA or its aminated metabolite. (a) Experimental design. (b) Body weight gain. (c) Disease activity index. (d) Representative picture and colon length. (e) Representative image of colons after hematoxylin and eosin staining (100× and 200×). The locations of crypts and goblet cells were marked with black and green arrows, respectively. (f) Serum fluorescein isothiocyanate-dextran content. Western blot image of (g) IL-4, IL-10, and TNF-α; (h) Akt, p-Akt, and PI3K; their relative expressions were normalized to the FMT-DSS group. Relative mRNA level of (i) NF-κB and COX-2; (j) PI3K and AKT; and (k) JNK, p38, and ERK. Data were represented as mean ± SD. ^#,^*P < 0.05, **P < 0.01, ^###,^***P < 0.001.

Then, we explored whether the modulated gut microbiota mediated the anti-colitis activity of GA and 4-NH_2_-GA via FMT (Fig. 5a). FMT from different donor mice had no impact on the water and food intake of the recipient mice (Fig. S7). FMT-GA and FMT-NGA mice exhibited improved colitis phenotypes, colonic histopathological changes, and intestinal permeability compared to FMT-DSS mice (Fig. 5b through f). Both FMT from GA and 4-NH_2_-GA elevated the protein expressions of IL-4 and IL-10, while FMT from 4-NH_2_-GA exhibited an additional reduction in the level of TNF-*α* (Fig. 5g). In addition, these transplantations resulted in a significant downregulation in the mRNA levels of colonic *tnf-α* and *il-1β* (Fig. S8). Moreover, compared to the mice from the FMT-DSS group, FMT from GA-treated donor mice significantly downregulated the mRNA levels of genes involved in NF-κB, PI3K/Akt, and MAPK signaling pathways, as well as reduced expression of PI3K and phosphorylation of Akt and Erk in the colon. In comparison, transplantation of 4-NH_2_-GA modulated microbiota showed similar and even slightly better inhibitory effects on activating these signaling pathways (Fig. 5h through l). Together, these data supported that the anti-colitis activity of GA and its aminated metabolite was mediated by gut microbiota.

### FMT treatment and compound supplementation modulated the gut microbiota in a different manner

**Fig 6:**
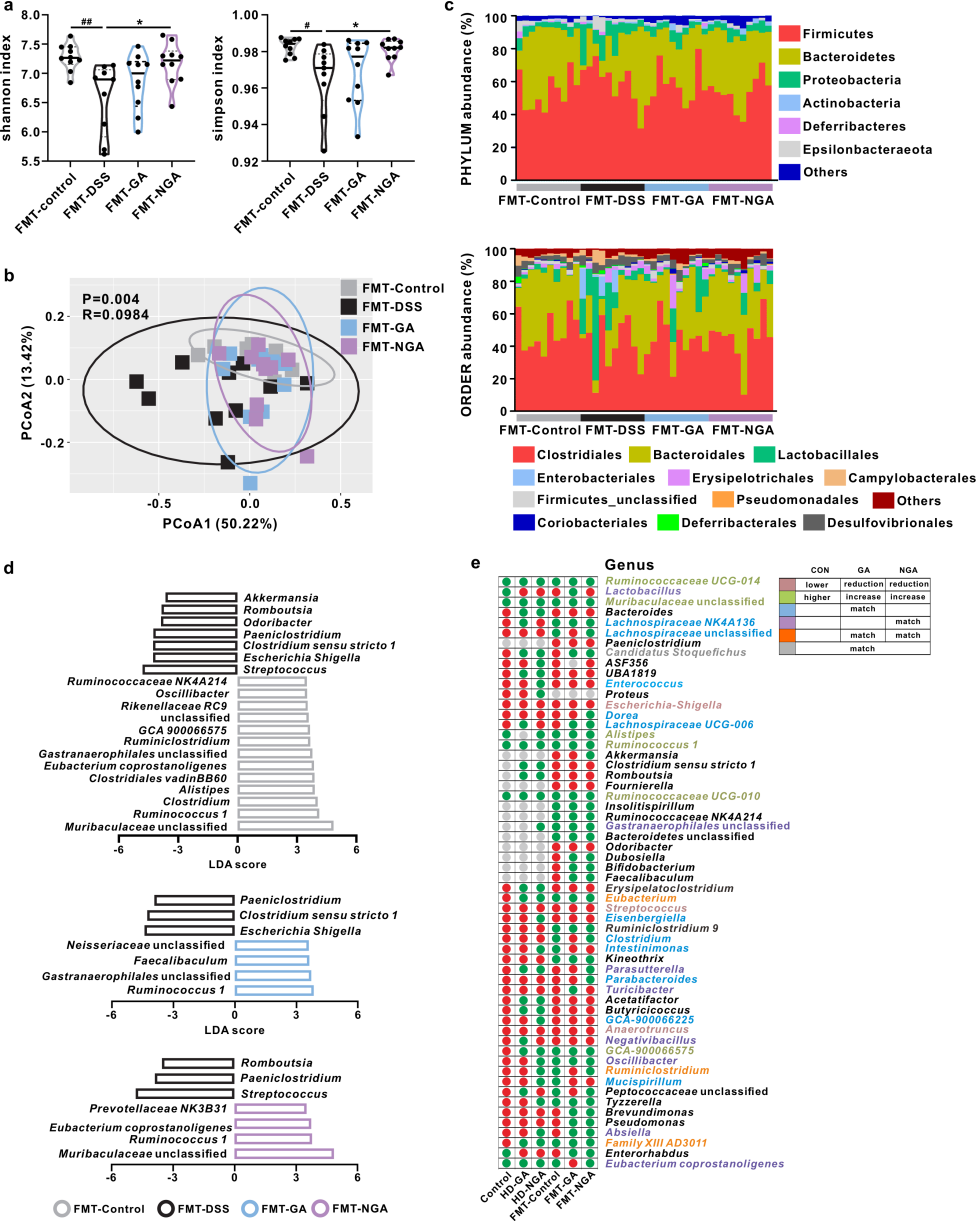
Gut microbiota modulation in FMT experiment. (a) α-Diversity (Shannon and Simpson indices). (b) UniFrac-based PCoA analysis. (c) Gut microbiota profile at phylum and order levels. (d) Biomarkers identified by linear discriminant analysis effect size analysis (*P* < 0.05, LDA >3.5 or <−3.5). (e) Comparison of the change in trend between the compound supplementation and FMT experiments. Green and red dots indicated increased and decreased abundance of key genera in the control, HD-GA, and HD-NGA groups compared to the DSS group. The same color rule was applied to the FMT-control, FMT-GA, and FMT-NGA groups, except the comparison was based on the FMT-DSS group. The color of each genus was marked according to its change trends between compound supplementation and FMT experiments. The deep pink color represents a trend where the level of genera was lower in the control group than in the DSS group, and both compounds reversed the increase brought about by DSS administration. The gray color marked the genera in which the trends in the control group differed from those in the treatment groups. The light green color represents the opposite trend. The remaining color represents genera that only showed a similar trend in one or two treatment groups. Data were represented as mean ± SD. ^#,^*P < 0.05, ^##^*P* < 0.01.

As shown in Fig. 6a; Fig. S6d, FMT from GA-treated (*P* > 0.05) and 4-NH_2_-GA-treated mice (*P* < 0.05) increased the α-diversity indices compared to the FMT-DSS group. PCoA showed a slight differentiation of FMT-DSS from the other three groups, indicating that the change induced by FMT was relatively milder than that caused by compound supplementation (Fig. 6b). Phylum-based phylogenetic analysis demonstrated similar yet distinguishable microbiota patterns between the FMT-GA and FMT-NGA groups when compared to the FMT-DSS group. The same trend in the change of Firmicutes and Bacteroidetes was observed in both diet treatments and the corresponding FMT treatments. At the order level, both FMT-GA and FMT-NGA treatments restored eight orders compared to the FMT-DSS group, indicating a more specific and beneficial modulation in gut microbiota achieved by FMT than the diet treatments (Fig. 6c; Table S4). In addition, the FMT-GA group was enriched in *Ruminococcus 1*, unclassified Gastranaerophilales, *Faecalibaculum* and unclassified Neisseriaceae, whereas the FMT-NGA group was enriched in unclassified Muribaculaceae, *Ruminococcus 1*, *Eubacterium coprostanoligenes*, and Prevotellaceae NK3B31 (Fig. 6d). Among 20 key genera identified in the FMT-treated groups, *Akkermansia* and *Eubacterium coprostanoligenes* were restored only by FMT-GA and FMT-NGA, respectively, while the other 15 genera were restored by both FMT treatments (Table S5). Spearman rank correlation analysis showed that seven and three genera were negatively and positively correlated with the severity of colitis (*P* < 0.05), respectively (Fig. S9). Among these 44 (compounds supplementation) and 20 (FMT treatment) key genera identified in this study, only 4 were repeatedly identified. Furthermore, three genera with a similar trend of modulation were observed, namely, *Eubacterium*, *Ruminiclostridium*, and *Family_XIII_AD3011_group* (Fig. 6e). These data indicated that although both compound supplementation and FMT treatments alleviated colitis, they modulated the gut microbiota in a different manner.

## DISCUSSION

In this study, the aminated product was detected in GA-administered mice and further identified as 4-NH_2_-GA by comparison with *in vitro* chemically synthesized standard. We found that the newly formed metabolite retained the original anti-colitis bioactivity of GA, and the anti-colitis activity of both GA and its aminated product was mediated by gut microbiota. These data provide new insights into the anti-colitis metabolism of GA and, more importantly, how polyphenols can exert various functions despite their low bioavailability.

Ammonia is considered to be one of the major sources of nitrogen for gut microbiota to synthesize amino acids, which contributes to the overall fecal amino acids levels that are positively correlated with increasing Crohn’s disease severity and gut microbiota dysbiosis, notably the blooming of many taxa from the Proteobacteria phylum, which has been commonly observed in IBD individuals (21, 22). Meanwhile, the over-expression of urease in *Escherichia coli*, a major bacterial enzyme responsible for the ammonia production, exacerbated colitis phenotypes in inoculated mice (23). Moreover, direct evidence of the harmful effects of ammonia has also been reported that ammonia exposure increased oxidative stress, which leads to an impaired tight junction protein barrier in intestinal epithelial cells and suppresses phagocytosis and immune response in dendritic cells (7, 8). Together, these reports suggested that ammonia acted as a harmful microbial metabolite during the development of IBD. Hence, reducing its level could be helpful in the maintenance of overall wellness. In this study, we demonstrated that GA could capture ammonia *in vivo* and form a new metabolite 4-NH_2_-GA (Fig. 1c), thus potentially reducing the level of free ammonia and its related toxicity while retaining or even slightly improving its original anti-colitis effect, as well as improved impaired intestinal permeability (Fig. 2b through h).

Being one of the simplest phenolic compounds with a specific skeleton, a *vic*-trihydroxyl group on the aromatic ring, the amination of GA implies that it could be a characteristic structure that can react with ammonia *in vivo.* The previous study suggested an essential role of gut microbiota in the amination of EGCG (16). In our study, the gut microbiota may mainly serve as a supplier of ammonia substrates instead of directly participating in the amination process. This hypothesis was supported by a decline in ammonia levels in the feces of antibiotic-treated mice (Fig. 1e and f). In addition, we also tried to increase luminal ammonia content by providing additional ammonium chloride to mice and detected the production of aminated GA *in vivo*. However, this method did not increase the colonic ammonia content as expected. Thus, further investigations are warranted to give us a more evident answer. Overall, our findings suggested that gut microbiota can participate in the *in vivo* amination of GA via direct supplementation with ammonia, which may also apply to other polyphenols containing this building block, thus providing an understanding of novel mechanisms in the metabolisms of polyphenols.

Although GA can reduce harmful microbial metabolite ammonia through amination, a more effective way to recover the dysbiosis in metabolites is to fix their source by improving the gut microbiota. In the present study, the gut microbiota modulation effects of GA and aminated GA were observed, along with improved colitis symptoms (Fig. 4). Generally, the gut microbiota is recognized as an essential mediator between environmental stimuli and the host immune system. By colonizing the mucosal interface, commensal bacteria form a natural barrier to invading potential pathogens. Communication between microbes and the immune system, either via metabolites (SCFAs) or direct presence, is core to maintaining immune homeostasis; otherwise, chronic intestinal inflammation or even further lesions would likely occur (24). In addition, the recovery of some bacteria commonly related to IBD amelioration was observed and vice versa. In line with previous studies that recorded a significant elevation of propionate and butyrate after intake of GA (25), most of those recovered bacteria in this study were reported as SCFA producers, including *Ruminococcus 1*, *Eubacterium*, Muribaculaceae, and *Alistipes* (26), which were enriched by GA and/or 4-NH_2_-GA treatments. GA and 4-NH_2_-GA also showed the ability to reduce genera that were pathobiont and most represented in UC patients, including *Parabacteroides*, *Streptococcus*, and *Escherichia-Shigella* (27). In addition, it has been reported that humans may not have hyper-ammonia-producing bacteria, while some pathogens can degrade amino acids to produce ammonia, and most of them belonged to Firmicutes and Proteobacteria (28), which showed reduced abundance after GA and 4-NH_2_-GA treatments.

However, even though we can see an improvement in the gut microbiota, it remains unclear whether gut microbes played a causative or a correlative role in the protection offered by these compounds, and it is difficult to recognize the potential microbiota-derived benefits. Therefore, we further conducted FMT in colitis mice and found alleviated colitis symptoms as expected (Fig. 5), indicating that modulated gut microbiota was able to protect the mice against colitis. It is worth mentioning that the supplementation with both compounds and FMT showed upregulation of the secretion of anti-inflammatory cytokines, especially on IL-10 (Fig. 2g, h, and 5g). Given that previous studies have already pointed out that therapeutic FMT controlled intestinal inflammation through IL-10 secretion by immune cells and microbiota-derived SCFAs promoted T helper 1 (Th1) cell IL-10 production to maintain intestinal homeostasis (29), such similarity may imply that a more robust suppression of the inflammatory response may be the critical factor for the anti-colitis effect of GA and aminated GA in which microbiota can be a significant contributor. After further analysis of the microbiota pattern in FMT recipient mice, we noticed that the FMT-control group had the highest α-diversity, while its microbial structure showed a distinct difference regarding the rest of the FMT groups (Fig. 6a). A more diverse microbiota community was observed in the control group than the FMT-GA and FMT-NGA groups, which might help to maintain sufficient colonization of the intestinal surface and to prevent invasion of pathogens and related mucosal lesions caused by provoked inflammatory response in the following FMT experiment (30). In addition, the overall microbiota from GA and aminated GA treatment groups might have already shifted toward a more specific anti-inflammatory profile under the prior challenge of DSS, thus performing better in regulating the secretion of cytokines regardless of lower diversity in the FMT-GA and FMT-NGA groups.

In addition, we noticed distinct differences in the enriched genera and gut microbiota composition between donor mice in dietary interventions and recipient mice in FMT treatments. Part of the reason can be attributed to the huge biological differences between different batches of mice, especially regarding their natural gut microbiota structure. However, it is also possible that even though most of the microbiota enriched in donor mice were not able to colonize the gut of recipient mice persistently, they could still convey their benefits as transient colonizers by promoting the growth of other beneficial microbes, competing with pathogens, or producing protective metabolites before they are flushed out (31). Together, these results demonstrate that GA and 4-NH_2_-GA treatment can alter the microbial profile toward a healthier one, and the beneficial effects of both GA and 4-NH_2_-GA against colitis are mediated by the gut microbiota, thus revealing a mutually interactive relationship between active compounds and gut microbiota, and providing further explanations on the ways through which low-bioavailability phytochemicals exert their therapeutic bioactivities.

In conclusion, a new mechanism was proposed for the anti-colitis effect of GA in this study. GA could reduce harmful microbial metabolite ammonia and form the new metabolite 4-NH_2_-GA, which retained its original anti-colitis function. In addition, GA can improve the gut microbiota, which mediates the protection against IBD. Considering GA structure is presented in various polyphenols as a common building block, our findings might provide a novel and universal understanding of the metabolism and action of polyphenols and diet-microbiota-host interactions.

## MATERIALS AND METHODS

### Chemical synthesis, purification, and characterization of 4-NH_2_-GA

GA (1.5 g; Sigma-Aldrich, St. Louis, MA, USA) was mixed with 50 mL of 25% ammonium hydroxide, and the solution was stirred for 70 min at room temperature. After the reaction was terminated by removing the excessive ammonium, the solution was analyzed by LC-MS to identify the new compound. Then, the solution was concentrated *in vacuo* and redissolved in ethanol. The new compound was purified by a silica gel column. The column was eluted with ethyl acetate (solvent A) and hexane (solvent B), starting with 20% A, followed by an increase to 40% A, 60% A, and finally to 80% A. The fractions collected were analyzed by high-performance liquid chromatography (HPLC), and those that contained the pure targeted compound were combined and concentrated. The purified product was identified by ^1^H- and ^13^C-NMR (solvent: dimethyl sulfoxide (DMSO)-*d6*; Bruker Avance 400-MHz NMR spectrometer, Billerica, MA, USA). The detailed methods of HPLC and LC-MS can be found in the supplimental material.

### Animal experiments

#### Animal and diet

C57BL/6J male mice (6–8 weeks old) were purchased from Zhejiang Ziyuan Laboratory Animal Technology Center Co. Ltd. (Hangzhou, Zhejiang, China). The mice were housed under a standard specific pathogen-free environment, with a 12:12-h light-dark cycle and a room temperature of 22°C. The mice had free access to water and a normal chow diet.

#### GA amination experiment

After acclimation for 1 week, the mice were divided into two groups (10 mice per group). One group of mice was supplied with water containing an antibiotic cocktail (40 mg/100 mL of metronidazole, penicillin, and neomycin, and 20 mg/100 mL of clindamycin and vancomycin), while the other group was administered with regular water. After 14 days, mice were fasted overnight and administered GA (200 mg/kg in 20% DMSO/water) by oral gavage. After 6 h, the fecal samples were collected, freeze-dried, mashed, and mixed. On the one hand, the fecal samples were extracted with 80% aqueous methanol, and the supernatant was analyzed by LC-MS. On the other hand, the fecal samples were extracted with distilled water, and the ammonia content was measured using a commercial kit (Nanjing Jiancheng Bioengineering Institute, Nanjing, Jiangsu, China).

#### Compound supplementation experiment

After acclimation for 1 week, the mice were divided into eight groups, with 10 mice per group. For the first 5 days, drinking water was given to the control group and DSS groups, while different concentrations of compound GA or 4-NH_2_-GA in drinking water at 0.002% (low-dose [LD] group), 0.01% (moderate-dose [MD] group), and 0.05% (high-does [HD] group) were administered to the left six treatment groups (32). DSS (Aladdin, Shanghai, China) was added to water for the following 10 days and reached a final concentration of 2.5% for all the groups except the control.

To assess the severity of colitis, the disease activity index (DAI) was recorded daily throughout the study. The DAI included three parameters: consistency of stool, blood in stool, and body weight loss. At the end of the experiment, the fecal samples were collected; four mice were selected to measure the intestinal membrane permeability via fluorescein isothiocyanate-dextran (Sigma-Aldrich), and the detailed method can be found in the supplimental material. After the mice were sacrificed, the colon length was measured; partial colon tissue (about 1 cm in length) was fixed with 10% formalin; and the remaining tissue was stored at −80°C for quantitative real-time PCR (qRT-PCR) and Western blot analysis. The detailed methods for qRT-PCR assay (primers are listed in Table S1) and Western blot assay can be found in the supplimental material.

#### FMT experiment

After acclimation for 1 week, the mice were divided into four groups with 10 mice per group. All mice were given drinking water for the first 5 days, followed by administering 2.5% DSS in drinking water for 10 days. For the entire study (15 days), these mice received 150 µL/mice/day FMT solution from the corresponding donor groups by oral gavage, respectively. The preparation of the FMT solution can be found in the supplimental material.

### Histological analysis

Formalin-fixed colon tissues were embedded in paraffin, sectioned, and stained with hematoxylin and eosin. The extent of histopathological alterations was evaluated under a microscope based on changes in crypt structure, crypt loss, and immune cell infiltration.

### Serum cytokine measurement

The concentrations of serum TNF-α, IL-1β, IL-4, and IL-10 were measured by enzyme-linked immunosorbent assay kits according to the manufacturer’s instructions (MultiSciences Biotech Co., Ltd., Shanghai, China).

### 16s rRNA gene sequencing

The 16S rRNA gene sequencing of colonic contents was performed in LC-Bio (Hangzhou, Zhejiang, China) with an Illumina MiSeq platform (Illumina, San Diego, CA, USA) using 2 × 300 bp paired-end (PE) sequencing. The software fqtrim (v.0.94), Vsearch (v.2.3.4), SILVA (release 132) classifier, QIIME2 (https://qiime2.org/), and OmicStudio tools (https://www.omicstudio.cn/tool) were used for data analysis. The detailed methods can be found in the supplimental material.

### Statistical analysis

Data are presented as in mean ± standard deviation. A two-tailed Student *t*-test was used to assess significant differences between the two groups and the DSS group with a significance threshold of *P* < 0.05.

## Data Availability

Raw sequence data are available and deposited at the National Center for Biotechnology Information Sequence Read Archive repository under the accession number PRJNA974546.

## SUPPLEMENTAL MATERIAL

Supplemental material mbio.02752-23-s0001.docx Supplemental text, Tables S1 to S5, and Fig. S1 to S9.

ASM does not own the copyrights to Supplemental Material that may be linked to, or accessed through, an article. The authors have granted ASM a non-exclusive, world-wide license to publish the Supplemental Material files. Please contact the corresponding author directly for reuse.
