# Supplementary material for: Gallic acid ameliorates colitis by trapping deleterious metabolite ammonia and improving gut microbiota dysbiosis
Source: mBio. 2023 Dec 21;15(2):e02752-23. doi: 10.1128/mbio.02752-23 (PMC10865988; doi:10.1128/mbio.02752-23)
Supplement: Supplemental material — Supplemental text, Tables S1 to S5, and Fig. S1 to S9. [file mbio.02752-23-s0001.docx]

*Supporting information for*

**Gallic acid ameliorates colitis by trapping deleterious metabolite ammonia and improving gut microbiota dysbiosis**

Jie Peng^2^, Tong Liu^1^, Pengfei Meng^1^, Yue Luo^2^, Siyue Zhu^2^, Yanxin Wang^1^, Mingxia Ma^1^, Jiaojiao Han^1^, Jun Zhou^1^, Xiurong Su^1^, Shiming Li^4^, Chi-Tang Ho^2^*, Chenyang Lu^1,3^*

^1^ State Key Laboratory for Managing Biotic and Chemical Threats to the Quality and Safety of Agro-products and School of Marine Science, Ningbo University, Ningbo 315211, China

^2^ Department of Food Science, Rutgers University, New Brunswick, New Jersey 08901, United States.

^3^ School of Food Science and Biotechnology, Zhejiang Gongshang University, Hangzhou 310018, China

^4^ College of Biology and Agricultural Resources, Huanggang Normal University, Huanggang 438000, China

*** Corresponding author**

**Dr. Chi-Tang Ho**

E-mail address: [ctho@sebs.rutgers.edu](mailto:ctho@sebs.rutgers.edu)

Postal address: Rutgers University, 65 Dudley Road, New Brunswick, NJ 08901, United States.

**Dr. Chenyang Lu**

E-mail address: luchenyang@nbu.edu.cn

Postal address: Ningbo University, 169 Qixing South Road, Ningbo 315211, China

**Materials and Methods**

**1. HPLC analysis**

Analysis of GA and aminated-GA were performed on an UltiMate 3000 HPLC system (ThermoFisher Scientific, Waltham, MA, USA) coupled with a DAD detector and a ZOEBAX Phenyl C18 column (250 x 4.6 mm, 5 µm, 100 Ǻ. Agilent, Santa Clara, CA, USA). Mobile phases A and B were water containing 0.1% formic acid and acetonitrile, respectively. The flow rate was set at 1 mL/min, and the mobile phase A was maintained at 98% for 15 min. The detection wavelength was 280 nm for both GA and aminated GA.

**2. LC-MS analysis**

LC-MS analysis of GA and aminated GA was performed on 1290 Infinity II/ 6545 QTOF LC/MS (Agilent, Santa Clara, CA, USA) in negative ion mode, and a TC-C18 column (250 x 4.6 mm, 5 μm, Agilent, Santa Clara, CA, USA) was used. For the LC, mobile phases A and B were water with 0.1% formic acid and acetonitrile, respectively, and the flow rate was set at 0.4 mL/min. Mobile phase A was maintained at 95% for the first 5 min, then gradually changed to 85% within 5 min, further decreased to 60% within 15 min, and finally to 0% in the last 5 min. The detection wavelength was set at 280 nm. For the mass spectroscopy, ionization parameters included nebulizer pressure (35 psi), drying gas flow (8 L/min), and temperature (320 °C). A full scan was performed between 100 and 1000 m/z. The elution time and m/z ratio (accurate to four decimal places) of the standard were determined, and the number was applied to the analysis of further samples using an extracted-ion chromatogram.

**3. Intestinal membrane permeability measurement**

At the end of the experiment, four mice were randomly selected and given FITC-dextran in phosphate-buffered saline (PBS) at 660 mg/kg body weight. Then, they were sacrificed after 4 h. Plasma was collected and centrifuged at 4 °C (3000 x rpm for 15 min) and was used to measure intestinal membrane permeability by fluorescence spectroscopy Infinite M200 Pro (Tecan, Männedorf, Switzerland). Fluorescence was measured at 530 nm with an excitation wavelength of 485 nm.

**4. RNA extraction, cDNA synthesis, and quantitative real-time PCR (qRT-PCR)**

Total RNA was extracted from the frozen colon sample with *TransZol* Up Plus RNA Kit (TransGen Biotech, Beijing, China) based on the manufacturer’s instructions. The extracted RNA was quantified by NanoDrop Spectrophotometer, and the quality of it was examined by measuring the absorbance at 260 nm and 280 nm (ThermoFisher, MA, USA). Reverse transcription of RNA was done by using TransScript®All-in-One First-Strand cDNA Synthesis SuperMix kit according to the manufacturer’s protocol (TransGen Biotech). Synthesized cDNA was diluted 100-fold, and 5 μl of each diluted cDNA was used for qPCR reactions (SYBR Green). After adding primers and premix for PCR, the final volume of the reaction system was brought up to 20 μl. The mRNA levels were normalized to β-actin and calculated using the 2^-∆∆CT^ method. Sequences of used primers were listed in Table S1.

**5. Western blot assay**

Total protein was extracted using RIPA buffer (Sangon Biotech Co., Ltd, Shanghai, China). The sample was homogenized with lysis buffer, and centrifugation at 12000 x rpm for 15 min. The supernatant was collected, and protein contents were measured by a bicinchoninic acid (BCA) protein assay kit (Solarbio, Beijing, China) according to the manufacturer’s instructions. Denatured proteins in samples were separated by SDS-PAGE and then transblotted onto a polyvinylidene difluoride (PVDF) membrane (Immun-Blot®, Bio-Rad Laboratories, Inc., Hercules, CA, USA). The membrane was blocked by 5% non-fat milk for 1 h at room temperature and then incubated with selected primary antibodies overnight at 4 °C. The membrane was washed with TBST three times before and after 1 h incubation with secondary antibodies at room temperature. Finally, chemiluminescence detection of protein bands was done by using the ChemiDoc XRS+ imaging system (Bio-Rad Laboratories Inc, Hercules, CA, USA) after applying WesternBright ECL kit (APGbio, Hongkong, China). The immunoblot bands were quantified by Image J software and further normalized to β-actin. Relative expression in other groups was normalized to the control group in the Compounds supplementation experiment or to the FMT-DSS group in the FMT experiment. The ratios of p-p38 to p38, p-Erk to Erk, and p-Akt to Akt were calculated and presented.

Antibodies against PI3K, JNK, p38, p-p38, p-Erk, Erk, and horse-radish peroxidase-conjugated secondary antibodies were purchased from Cell Signaling Technology (Danvers, MA, USA). Antibodies against COX-2, Akt, p-Akt, and β-actin were purchased from Sangon Biotech Co., Ltd. Antibodies against IL-4, IL-10, and TNF-α were purchased from Affinity Biosciences (Santa Barbara, CA, USA), Proteintech (Rosemont, IL, USA), ABclonal (Woburn, MA, USA) respectively.

**6. FMT solution preparation**

In the compound supplementation experiment, feces collected from the Control, DSS, HD-GA, and HD-NGA groups were homogenized, and sterile PBS was added to dilute the samples. The fecal solution was centrifuged at 1000 x rpm for 5 min. The supernatant was collected and centrifugated at 12000 x rpm for 10 min. After discarding the supernatant, the pellet was washed with PBS and centrifuged under the same condition. This process was repeated three times, and the washed pellet was resuspended in 20% glycerol solution, reaching a final concentration of 33 mg feces/ml. The solution was stored at -80°C for the FMT experiment.

**7. 16S rRNA gene sequencing**

The genomic DNA was extracted from colonic content using an E.Z.N.A. ® Stool DNA Kit (D4015, Omega, Inc., Auburn, WA, USA) according to the manufacturer’s instructions. Eluted genomic DNA was stored at -80 °C for measurement in the PCR conducted by LC-Bio Technology Co., Ltd (Hang Zhou, Zhejiang, China). The 5’-ends of the primers were tagged with specific barcodes for each sample and sequencing universal primers. The primers used were V3-V4 region primers 341F (5'-CCTACGGGNGGCWGCAG-3') and 805R (5'-GACTACHVGGGTATCTAATCC-3'). To prepare the reaction system, for 25 ng of template DNA from each sample, a 12.5 μl PCR premix was added, followed by 2.5 μl of each primer, and the total volume was brought up to 20 μl with PCR-grade water. The program of reaction was set as follows: the temperature was held at 98 °C for 30 s, followed by 32 cycles of 98 °C for 10 s, annealing at 54 °C for 30 s, and extension at 72 °C for 45 s, and finally ending with an additional extension at 72 °C for 10 min. Acquired PCR products were confirmed by 2% agarose gel electrophoresis, purified, and quantified using AMPure XT beads (Beckman Coulter Genomics, Danvers, MA, USA) and Qubit (Invitrogen, Waltham, MA, USA), respectively. The sequencing of libraries was performed on an Illumina MiSeq platform (Illumina, San Diego, CA, USA) using 2 × 300 bp pair-end sequencing. Quality filtering of the raw reads was performed using fqtrim (v0.94), whereas chimeric sequences were filtered using Vsearch software (v2.3.4). After dereplication, the DADA2 package was used to acquire the feature tables and sequences. α- and β-diversity were calculated by normalizing them to the same sequences randomly, feature abundance was normalized based on the relative abundance of each sample according to the SILVA (release 132) classifier, and α- and β-diversity indices were calculated using QIIME2. Sequence alignment was performed via BLAST, and the obtained feature sequence was annotated using the SILVA database for each representative sequence. The gut microbial structure was analyzed using UniFrac-based principal coordinates analysis (PCoA). LEfSe (*p*<0.05, LDA>4 or <-4), spearman correlation analysis, and advanced heatmap plots were performed using the OmicStudio tools (https://www.omicstudio.cn/tool).

**Table Legends**

**Table S1.** Primer sequences for target genes.

**Table S2.** Gut microbial profile at phylum and order levels in compounds supplementation experiment.

**Table S3.** Forty-four key genera of gut microbiota were identified in the compound supplementation experiment.

**Table S4.** Gut microbial profile at phylum and order levels in FMT experiment.

**Table S5.** Twenty key genera of gut microbiota were identified in the FMT experiment.

**Table S1.** Primer sequences for target genes.

| Target genes | Primer sequences |
| --- | --- |
| Actin | Forward: 5’-TGTCCACCTTCCAGCAGATG-3’  Reverse: 5’-GCTCAGTAACAGTCCGCCTA-3’ |
| NF-κB | Forward: 5’- AGCACAGATACCACCAAGACA -3’  Reverse: 5’- TCAGCCTCATAGTAGCCATCC -3’ |
| COX2 | Forward: 5’-GTGCGACATACTCAAGCAGG-3’  Reverse: 5’-TCAGGTGTTGCACGTAGTCT-5’ |
| iNOS | Forward: 5’- CAGCGGAGTGACGGCAAACA -3’  Reverse: 5’- CAAGACCAGAGGCAGCACATCA -5’ |
| PI3K | Forward: 5’-CGCATCAGCAAGACTCCAGAA-3’  Reverse: 5’-AGAACTCCGCAGCCACACT-3’ |
| Akt | Forward: 5’-TGACCATGAACGAGTTTGAGTA-3’  Reverse: 5’-GAGGATCTTCATGGCGTAGTAG-3’ |
| JNK | Forward: 5’- CGGACAAGCAGTTAGATGAGAGG-3’  Reverse: 5’- AGACGGCGAAGACGATGGAT-3’ |
| ERK1 | Forward: 5’-TCCAAGGGCTACACCAAATC-3’  Reverse: 5’-GTTTTCGAGGGCAGAGACTG-3’ |
| ERK2 | Forward: 5’-TACACCAACCTCTCGTACATCG-3’  Reverse: 5’-CATGTCTGAAGCGCAGTAAGATT-3’ |
| p38 | Forward: 5’-GACACCCCCTGCTTATCTCA-3’  Reverse: 5’-GGGCTGCTGTGATCCTCTTA-5’ |
| NLRP3 | Forward: 5’- GCTCAGGACATACGTCTGGA-3’  Reverse: 5’- TGAGGTCCACATCTTCAAGG-3’ |
| ASC | Forward: 5’-GTGCTTAGAGACATGGGCTTAC-3’  Reverse: 5’-GTTCTGGCTGTACTCTGAGCA-3’ |
| Caspase1 | Forward: 5’-GGACCCTCAAGTTTTGCCCT-3’  Reverse: 5’-AACTTGAGCTCCAACCCTCG-5’ |
| TLR4 | Forward: 5’-GGACTATGTGATGTGACCATTGAT-3’  Reverse: 5’-TTATAGATACACCTGCCAGAGACA-3’ |
| MyD88 | Forward: 5’-CCGCATGGTGGTGGTTGTT-3’  Reverse: 5’-GGAATCAGTCGCTTCTGTTGGA-3’ |
| IL-4 | Forward: 5’-CCATATCCACGGATGCGACAA-3’  Reverse: 5’-TGGTGTTCTTCGTTGCTGTGA-5’ |
| IL-10 | Forward: 5’-CTGCTAACCGACTCCTTAATGC-3’  Reverse: 5’-GCTCCACTGCCTTGCTCTT-5’ |
| TNF-α | Forward: 5’- GTGGTGCCAGCCGATGGGTT-3’  Reverse: 5’- CTGCCCGGACTCCGCAAAGTC-3’ |
| IL-1β | Forward: 5’- ACAGCAGCACATCAACAAGAG-3’  Reverse: 5’- CCAGCAGGTTATCATCATCATCC-3’ |

**Table S2.** Gut microbial profile at phylum and order levels in compounds supplementation experiment.

| **PHYLUM** | Control | DSS | HD-GA | HD-NGA |
| --- | --- | --- | --- | --- |
| Firmicutes | 65.11±17.38 | 67.18±12.07 | 64.26±16.92 | 56.9±21.35 |
| Bacteroidetes | 33.43±16.74 | 21.32±13.78 | 27.31±18.39 | 31.18±22.31 |
| Proteobacteria | 0.21±0.19 | 10.43±8.15 | 7.49±10.58 | 10.63±7.56 |
| Actinobacteria | 1.07±0.77 | 0.29±0.15 | 0.23±0.11 | 0.13±0.08 |
| Deferribacteres | 0.02±0.03 | 0.62±0.49 | 0.33±0.24 | 0.62±0.59 |
| Others | 0.17±0.14 | 0.16±0.1 | 0.37±0.22 | 0.55±0.43 |
| **ORDER** | Control | DSS | HD-GA | HD-NGA |
| Clostridiales | 16.23±12.19 | 42.79±15.67 | 41.2±22.86 | 43.96±22.61 |
| Bacteroidales | 33.43±16.74 | 21.32±13.78 | 27.31±18.39 | 31.13±22.34 |
| Lactobacillales | 48.46±25.88 | 21.14±15.79 | 10.43±8.06 | 8.54±7.93 |
| Enterobacteriales | 0.01±0.03 | 8.68±8.29 | 6.28±9.99 | 8.87±7.2 |
| Erysipelotrichales | 0.09±0.07 | 2.61±1.58 | 12.09±12.39 | 3.93±3.76 |
| Firmicutes_unclassified | 0.33±0.28 | 0.61±0.28 | 0.55±0.54 | 0.46±0.37 |
| Pseudomonadales | 0.14±0.13 | 1±0.69 | 0.31±0.24 | 0.43±0.6 |
| Coriobacteriales | 1.07±0.77## | 0.26±0.13 | 0.17±0.09 | 0.08±0.03 |
| Deferribacterales | 0.02±0.03## | 0.62±0.49 | 0.33±0.24 | 0.62±0.59 |
| Others | 0.23±0.16### | 0.97±0.58 | 1.35±0.61 | 2±1.46 |

**Table S3.** Forty-four key genera of gut microbiota were identified in the compound supplementation experiment.

|  | Control | DSS | HD-GA | HD-NGA |
| --- | --- | --- | --- | --- |
| *Ruminococcus_1* | 0.42±0.43 | 0 | 0.01±0.03 | 0.02±0.05 |
| *Alistipes* | 1.67±0.99 | 0 | 0 | 0.13±0.2 |
| *Escherichia-Shigella* | 0.01±0.02 | 8.29±8.28 | 5.76±9.52 | 6.63±7.56 |
| *Mucispirillum* | 0.02±0.03 | 0.62±0.49 | 0.32±0.24 | 0.62±0.59 |
| *Dorea* | 0.01±0.01 | 1.43±1.43 | 0.43±0.27 | 0.61±0.66 |
| *Turicibacter* | 0 | 0.73±0.61 | 0.39±0.28 | 0.58±0.68 |
| *Parabacteroides* | 0 | 4.27±4.51 | 0.58±0.79 | 1.23±1.07 |
| *Lachnospiraceae_UCG-006* | 0.13±0.11 | 1.02±0.49 | 1.1±1.1 | 0.63±0.73 |
| *Oscillibacter* | 0.1±0.21 | 0.5±0.49 | 0.38±0.26 | 0.53±0.51 |
| *Ruminiclostridium_9* | 0.15±0.26 | 1.27±1.16 | 0.98±0.64 | 1.1±0.76 |
| *Anaerotruncus* | 0.07±0.06 | 0.4±0.32 | 0.36±0.33 | 0.21±0.23 |
| *Brevundimonas* | 0.04±0.04 | 0.26±0.2 | 0.15±0.1 | 0.2±0.28 |
| *Pseudomonas* | 0.14±0.13 | 1±0.69 | 0.31±0.24 | 0.42±0.59 |
| *Negativibacillus* | 0.03±0.05 | 0.16±0.21 | 0.31±0.22 | 0.12±0.14 |
| *GCA-900066575* | 0.06±0.08 | 0.17±0.15 | 0.25±0.19 | 0.48±0.41 |
| *Ruminiclostridium* | 0.06±0.07 | 0.26±0.21 | 0.25±0.26 | 0.51±0.44 |
| *GCA-900066225* | 0.01±0.01 | 0.21±0.15 | 0.18±0.12 | 0.31±0.29 |
| *Tyzzerella* | 0.04±0.07 | 0.28±0.19 | 0.23±0.3 | 0.59±0.44 |
| *unclassified* | 0.05±0.06 | 0.04±0.04 | 0.14±0.13 | 0.31±0.42 |
| *Proteus* | 0 | 0.23±0.43 | 0.14±0.16 | 2.16±2.25 |
| *UBA1819* | 0 | 0.13±0.17 | 0.19±0.15 | 0.24±0.33 |
| *Parasutterella* | 0.01±0.01 | 0.06±0.05 | 0.32±0.39 | 0.29±0.39 |
| *Family_XIII_AD3011_group* | 0.01±0.01 | 0.22±0.23 | 0.37±0.32 | 0.61±0.82 |
| *Absiella* | 0.02±0.02 | 0.14±0.2 | 0.09±0.07 | 0.19±0.21 |
| *Enterococcus* | 0 | 0.17±0.21 | 0.14±0.16 | 0.39±0.78 |
| *Candidatus_Stoquefichus* | 0 | 0.09±0.16 | 0.75±0.63 | 0.16±0.26 |
| *Kineothrix* | 0.03±0.09 | 0.66±0.97 | 0.45±0.56 | 0.15±0.14 |
| *ASF356* | 0.03±0.08 | 0.12±0.15 | 0.07±0.04 | 0.25±0.42 |
| *Acetatifactor* | 0.04±0.12 | 0.17±0.19 | 0.34±0.44 | 0.21±0.2 |
| *Butyricicoccus* | 0.01±0.02 | 0.27±0.42 | 0.64±0.76 | 0.33±0.34 |
| *Bacteroides* | 1.69±1.55 | 13.93±8.93 | 16.61±10.12 | 14.42±13.59 |
| *Muribaculaceae_unclassified* | 30.04±14.87 | 3.1±2.3 | 10.04±9.93 | 15.31±16.25 |
| *Lactobacillus* | 48.3±25.87 | 15.94±13.33 | 7.61±6.27 | 6.3±6.77 |
| *Enterorhabdus* | 1.07±0.76 | 0.25±0.12 | 0.16±0.09 | 0.08±0.03 |
| *Ruminococcaceae_UCG-014* | 3.2±1.96 | 0.34±0.37 | 1.11±1.39 | 0.68±0.8 |
| *Eubacterium* | 0.13±0.15 | 1.78±2.55 | 5.84±5.46 | 3.23±2.96 |
| *Peptococcaceae_unclassified* | 0.12±0.12 | 0.76±0.83 | 1.16±0.6 | 0.7±0.29 |
| *Erysipelatoclostridium* | 0.07±0.06 | 1.65±1.51 | 10.83±11.96 | 2.98±3.76 |
| *Intestinimonas* | 0.38±0.42 | 2.45±2 | 2.32±2.05 | 2.86±2.82 |
| *Clostridium* | 0.63±1.08 | 3.16±3.46 | 2.78±3.68 | 2.17±2.15 |
| *Lachnospiraceae_NK4A136_group* | 1.88±1.1 | 5.43±2.92 | 5.46±6.06 | 4.3±2.58 |
| *Lachnospiraceae_unclassified* | 1.43±1.67 | 8.47±5.88 | 5.47±5.27 | 7.55±6.45 |
| *Streptococcus* | 0.17±0.12 | 5.01±3.87 | 2.67±2.8 | 1.81±1.54 |
| *Eisenbergiella* | 0.16±0.17 | 5.67±2.89 | 5.53±4.32 | 6.83±6.21 |

**Table S4.** Gut microbial profile at phylum and order levels in FMT experiment.

| **PHYLUM** | FMT-Control | FMT-DSS | FMT-GA | FMT-NGA |
| --- | --- | --- | --- | --- |
| Firmicutes | 53.94±10.94 | 59.86±12.77 | 57.59±12.18 | 53.39±10.28 |
| Bacteroidetes | 35.97±13.52 | 24.51±17.34 | 31.4±11.82 | 35.84±9.12 |
| Proteobacteria | 4.35±1.61 | 10.19±7.26# | 4.84±2.3* | 4.86±1.76* |
| Actinobacteria | 0.42±0.35 | 0.47±0.37 | 1.03±1.12 | 1.23±1.14 |
| Deferribacteres | 0.63±1.24 | 0.55±0.69 | 0.32±0.59 | 0.12±0.13 |
| Epsilonbacteraeota | 2.15±1.69 | 2.87±3.02 | 1.13±1.1 | 0.99±0.75 |
| Others | 2.54±0.79 | 1.55±0.81# | 3.69±1.41*** | 3.58±2** |
| **ORDER** | FMT-Control | FMT-DSS | FMT-GA | FMT-NGA |
| Clostridiales | 49.11±12.15 | 40.6±16.03 | 43.57±12.51 | 44.82±14.77 |
| Bacteroidales | 35.75±13.48 | 24.4±17.29 | 31.26±11.77 | 35.68±9.11 |
| Lactobacillales | 2.07±2.44 | 15.74±19.3# | 7.17±9.75 | 3.59±4.33 |
| Enterobacteriales | 0.01±0.01 | 4.61±7.25 | 0.01±0.01 | 0.02±0.03 |
| Erysipelotrichales | 1.17±0.94 | 2.56±2.58 | 4.89±5.74 | 3.64±4.12 |
| Firmicutes_unclassified | 1.51±0.5 | 0.94±0.58# | 1.89±1.56 | 1.29±0.87 |
| Pseudomonadales | 0.03±0.02 | 0.08±0.08 | 0.11±0.16 | 0.12±0.17 |
| Coriobacteriales | 0.31±0.24 | 0.3±0.3 | 0.64±0.96 | 0.74±0.67 |
| Deferribacterales | 0.63±1.24 | 0.55±0.69 | 0.32±0.59 | 0.12±0.13 |
| Desulfovibrionales | 3.35±1.59 | 4.29±1.91 | 3.32±1.84 | 3.13±2.08 |
| Campylobacterales | 2.15±1.69 | 2.87±3.02 | 1.13±1.09 | 0.99±0.75 |
| Others | 3.91±1.13 | 3.07±1.83 | 5.68±2.41* | 5.85±2.9* |

**Table S5.** Twenty key genera of gut microbiota were identified in the FMT experiment.

|  | FMT-Control | FMT-DSS | FMT-GA | FMT-NGA |
| --- | --- | --- | --- | --- |
| *Paeniclostridium* | 0±0 | 1.5±2.35 | 0±0 | 0±0 |
| *Muribaculaceae_unclassified* | 23.83±10.6 | 10.6±9.17 | 18.24±6.57 | 22.89±6.89 |
| *Akkermansia* | 0.02±0.03 | 0.41±0.67 | 0.14±0.34 | 0.95±1.91 |
| *Bifidobacterium* | 0.09±0.1 | 0.13±0.13 | 0.32±0.23 | 0.44±0.48 |
| *Faecalibaculum* | 0.1±0.19 | 0.23±0.29 | 1.31±1.97 | 0.34±0.32 |
| *Odoribacter* | 0.24±0.28 | 1.31±1.97 | 0.32±0.35 | 0.3±0.21 |
| *Streptococcus* | 0.17±0.21 | 11.97±19.71 | 0.81±0.91 | 0.31±0.32 |
| *Romboutsia* | 0.03±0.02 | 0.85±1.1 | 0.73±1.36 | 0.15±0.11 |
| *Clostridium_sensu_stricto_1* | 0.06±0.05 | 3.56±5.11 | 0.16±0.22 | 0.69±0.95 |
| *Gastranaerophilales_unclassified* | 0.87±0.65 | 0.24±0.27 | 1.11±1.22 | 0.77±0.9 |
| *Bacteroidetes_unclassified* | 0.23±0.09 | 0.11±0.07 | 0.14±0.09 | 0.15±0.06 |
| *unclassified* | 0.69±0.29 | 0.19±0.16 | 0.65±0.51 | 0.38±0.21 |
| *Ruminococcus_1* | 1.8±0.87 | 0.28±0.33 | 1.64±1 | 1.54±1.43 |
| *Alistipes* | 1.11±0.59 | 0.56±0.33 | 1.02±0.46 | 1.04±0.67 |
| *Dubosiella* | 0.38±0.32 | 0.91±1.17 | 1.75±1.66 | 2.02±2.41 |
| *Insolitispirillum* | 0.2±0.17 | 0.05±0.12 | 0.24±0.38 | 0.11±0.15 |
| *Fournierella* | 0.29±0.14 | 0.34±0.4 | 0.02±0.03 | 0.28±0.37 |
| *Eubacterium_coprostanoligenes_group* | 0.95±1.29 | 0.17±0.45 | 0.15±0.11 | 1.3±1.31 |
| *Ruminococcaceae_UCG-010* | 0.14±0.08 | 0.04±0.05 | 0.14±0.07 | 0.15±0.1 |
| *Ruminococcaceae_NK4A214_group* | 0.35±0.18 | 0.05±0.07 | 0.25±0.22 | 0.15±0.2 |

**Figures Legends**

**Fig. S1** Structure of Myricetin, 4’-NH_2_-Myricetin, EGCG, and 4’-NH_2_-EGCG. The characteristic structure of *vic*-trihydroxyl group is highlighted in the red circle.

**Fig. S2** The NMR spectrum of GA and its aminated metabolite 4-NH_2_-GA. **(a)** ^13^C-NMR and **(b)** ^1^H-NMR spectrum of GA. **(c)** ^13^C-NMR and **(d)** ^1^H-NMR spectrum of 4-NH_2_-GA.

**Fig. S3** Detection of GA and its aminated metabolite (4-NH_2_-GA) in normal mice. **(a)** LC-MS spectrum of 4-NH2-GA and GA in fecal samples collected after oral gavage of GA in normal mice. **(b)** Peak areas of GA and 4-NH2-GA. Data was represented as mean ± SD.

**Fig. S4** The anti-colitis activity of different dosages of GA and its aminated metabolite in DSS-induced colitis mice. **(a)** Experimental design. **(b)** Water intake. **(c)** Food intake. **(d)** Disease activity index. **(e)** Colon length. **(f)** Serum FITC-dextran content. Data was represented as mean ± SD. #, * *p*<0.05, ### *p*<0.001.

**Fig. S5** The effects of GA and its aminated metabolite on serum cytokine levels in DSS-induced colitis mice. **(a)** IL-4. **(b)** IL-10. **(c)** TNF-α. **(d)** IL-1β. **(e)** Protein expression level of IL-4 and IL-10 in colonic tissue. **(f)** mRNA levels of TLR4 and MyD88. Data was represented as mean ± SD. #, * *p*<0.05, ** *p*<0.01, ***, ### *p*<0.001.

**Fig. S6** α-diversity of gut microbiota in compounds supplementation and FMT experiments. Observed OTUs index in **(a)** compounds supplementation and **(c)** FMT experiments. Chao1 index in **(b)** compounds supplementation and **(d)** FMT experiment. Data was represented as mean ± SD. # *p*<0.05, *** *p*<0.001.

**Fig. S7** Water intake and food intake of each group in the FMT experiment. Data was represented as mean ± SD.

**Fig. S8** mRNA levels of key genes involved in signaling pathways in FMT experiment. **(a)** mRNA levels of IL-4 and IL-10 and **(b)** TNF-α and IL-1β. **(c)** mRNA expression level of NLRP3, ASC and Caspase1. **(d)** mRNA expression level of TLR4 and MyD88. Data was represented as mean ± SD. *, # *p*<0.05, **, ## *p*<0.01.

**Fig. S9** Heatmap of 20 key genera responding to FMT treatments and correlation study between gut microbiota and colitis-related indexes. * *p*<0.05, ** *p*<0.01.


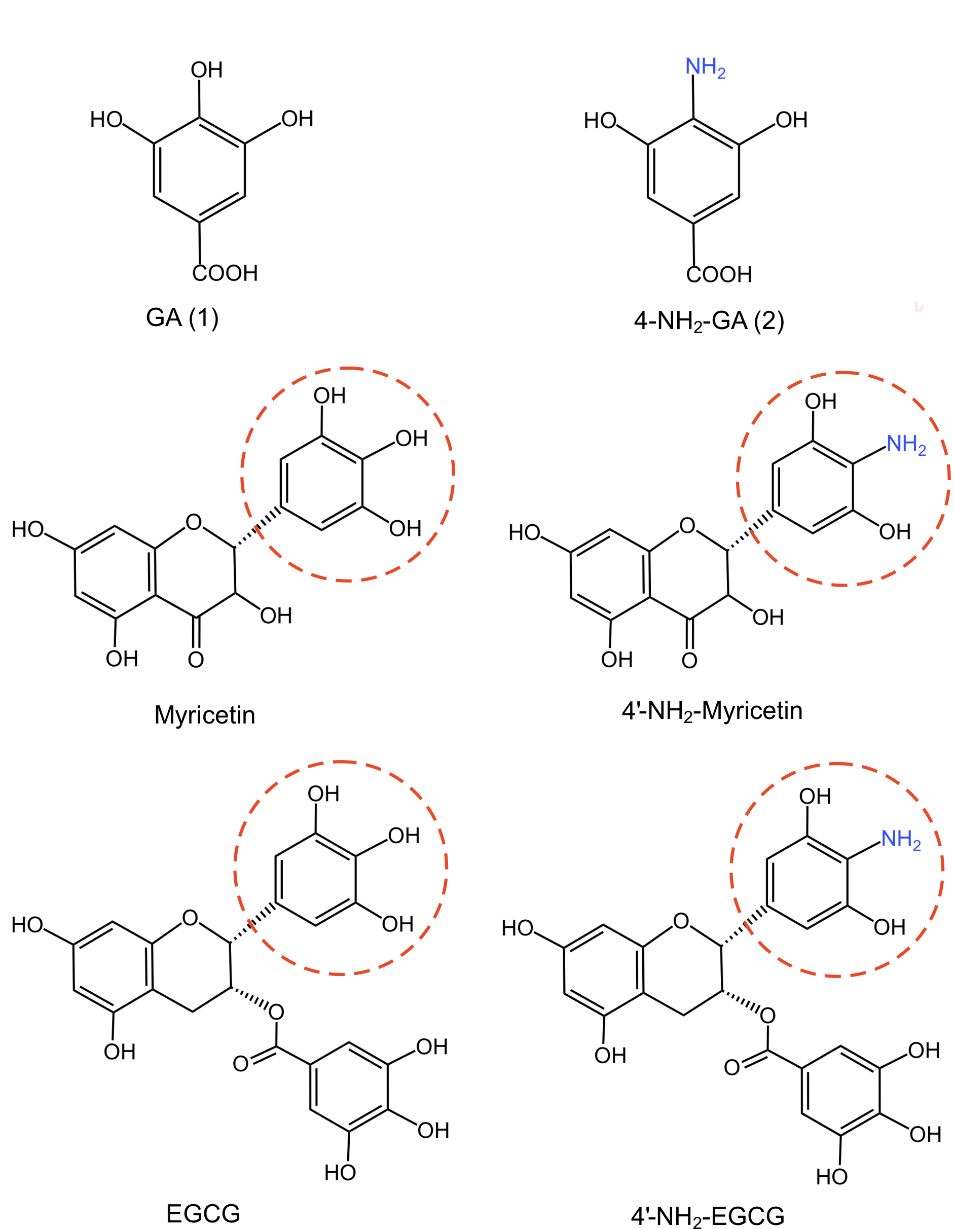


**Fig. S1** Structure of Myricetin, 4’-NH_2_-Myricetin, EGCG, and 4’-NH_2_-EGCG. The characteristic structure of *vic*-trihydroxyl group is highlighted in the red circle.

**
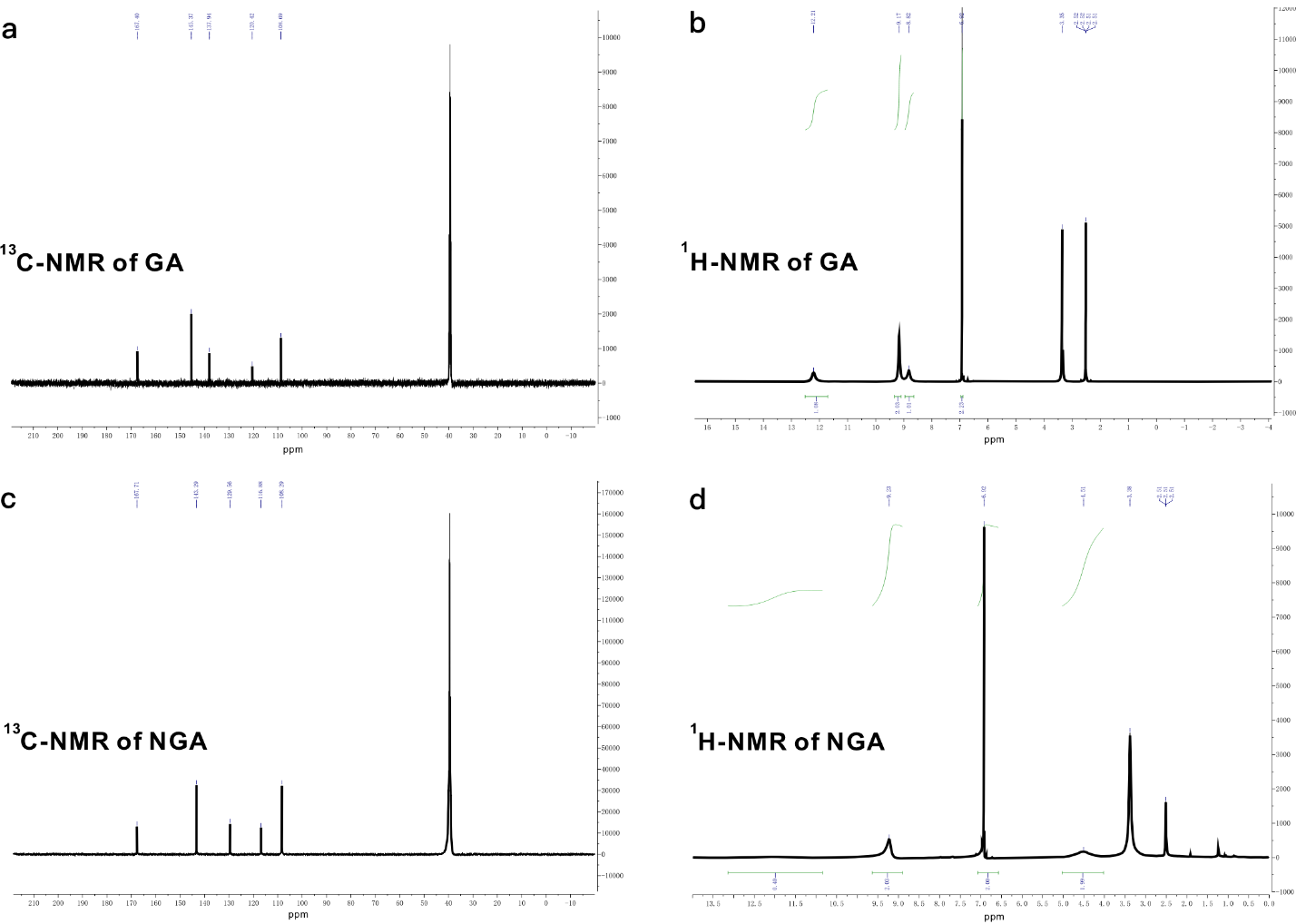
 Fig. S2** The NMR spectrum of GA and its aminated metabolite 4-NH_2_-GA. **(a)** ^13^C-NMR and **(b)** ^1^H-NMR spectrum of GA. **(c)** ^13^C-NMR and **(d)** ^1^H-NMR spectrum of 4-NH_2_-GA.


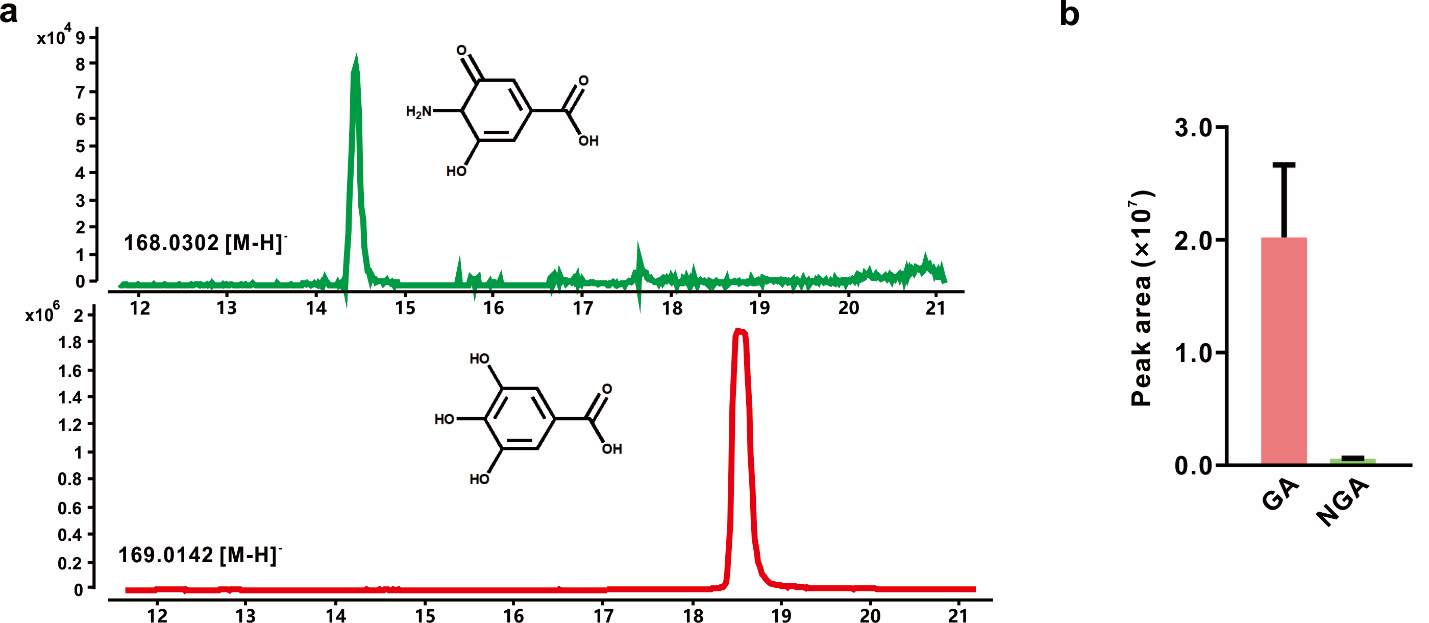


**Fig. S3** Detection of GA and its aminated metabolite (4-NH_2_-GA) in normal mice. **(a)** LC-MS spectrum of 4-NH2-GA and GA in fecal samples collected after oral gavage of GA in normal mice. **(b)** Peak areas of GA and 4-NH2-GA. Data was represented as mean ± SD.


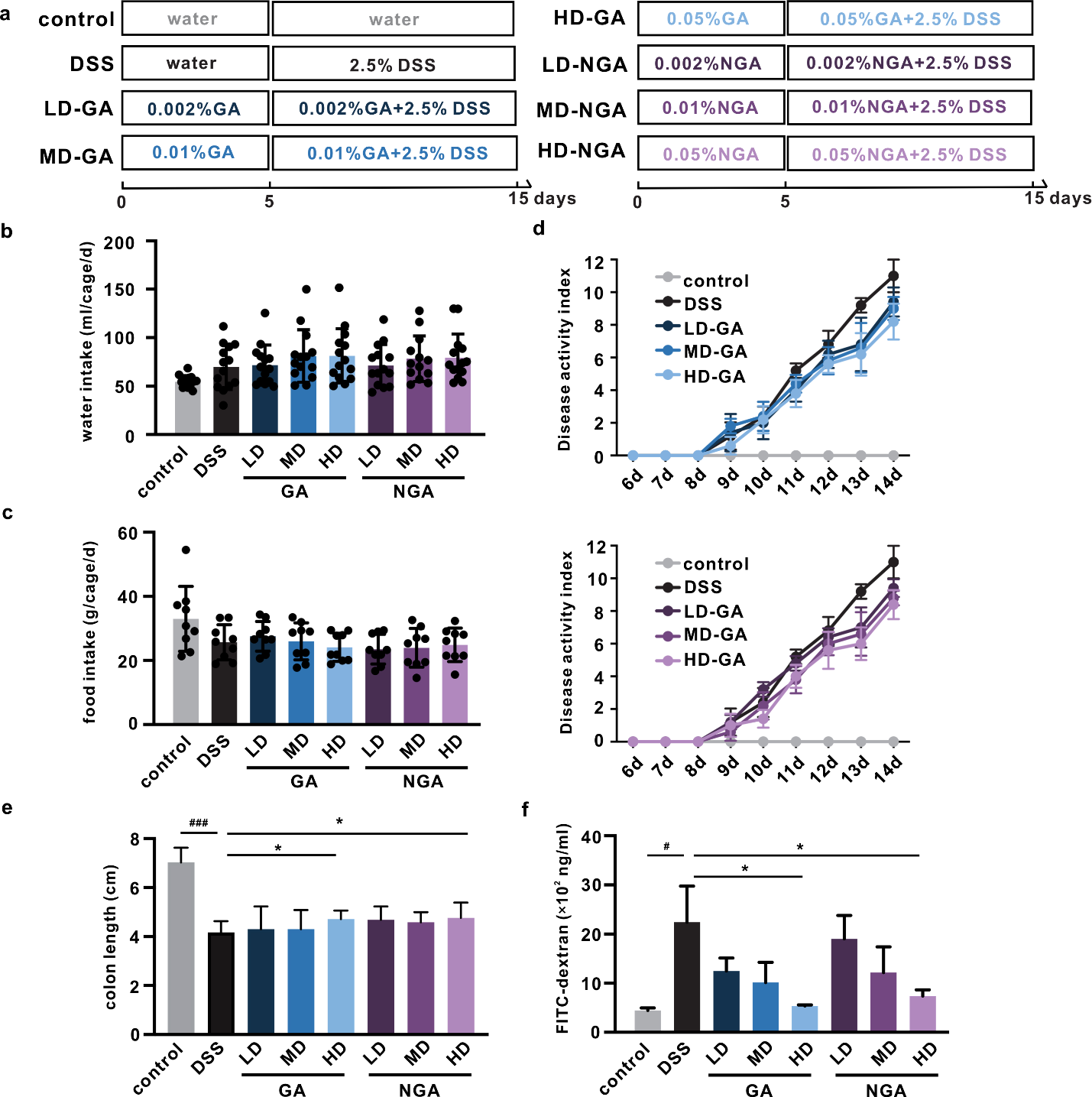


**Fig. S4** The anti-colitis activity of different dosages of GA and its aminated metabolite in DSS-induced colitis mice. **(a)** Experimental design. **(b)** Water intake. **(c)** Food intake. **(d)** Disease activity index. **(e)** Colon length. **(f)** Serum FITC-dextran content. Data was represented as mean ± SD. #, * *p*<0.05, ### *p*<0.001.


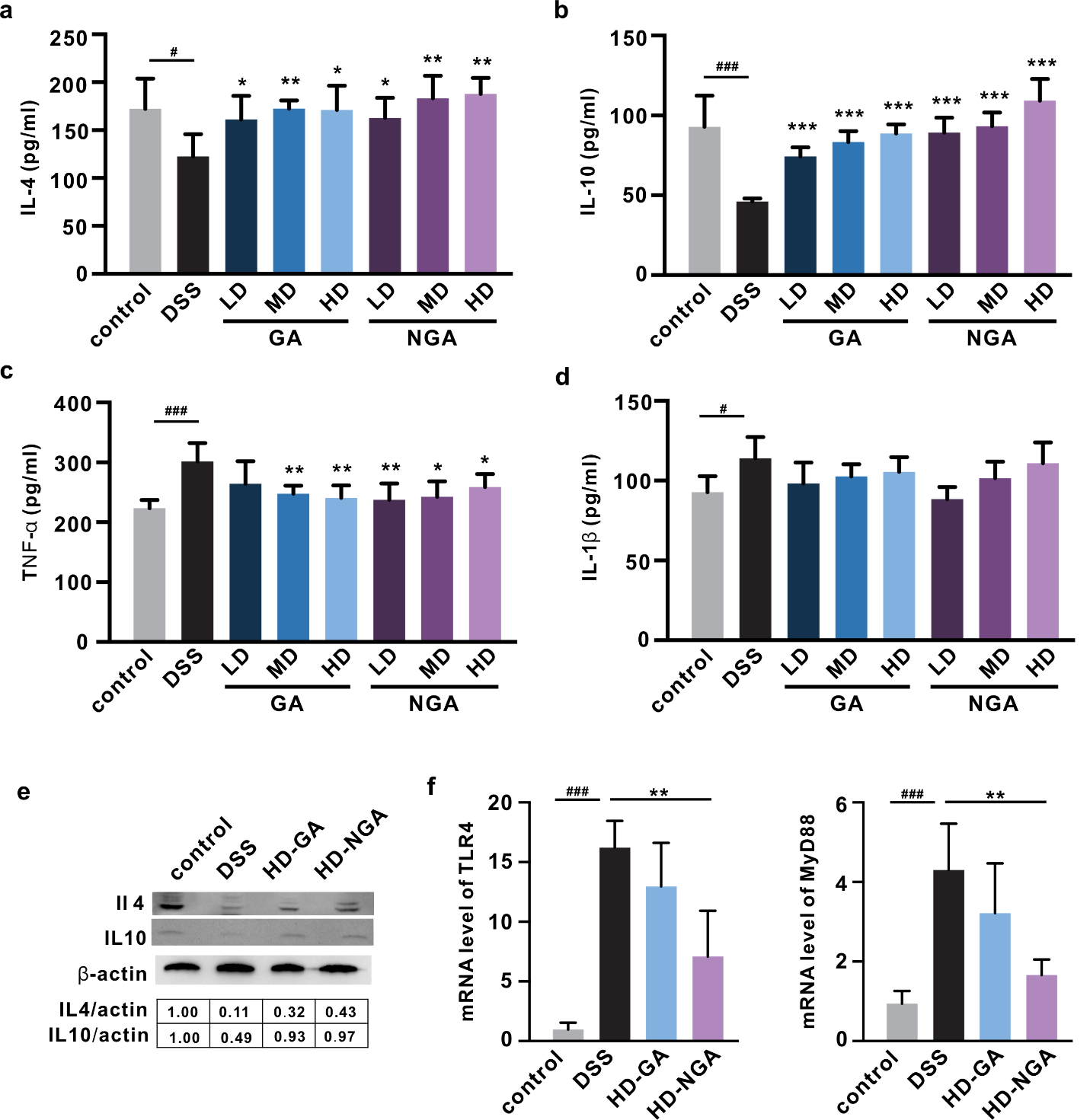


**Fig. S5** The effects of GA and its aminated metabolite on serum cytokine levels in DSS-induced colitis mice. **(a)** IL-4. **(b)** IL-10. **(c)** TNF-α. **(d)** IL-1β. **(e)** Protein expression level of IL-4 and IL-10 in colonic tissue. **(f)** mRNA levels of TLR4 and MyD88. Data was represented as mean ± SD. #, * *p*<0.05, ** *p*<0.01, ***, ### *p*<0.001.


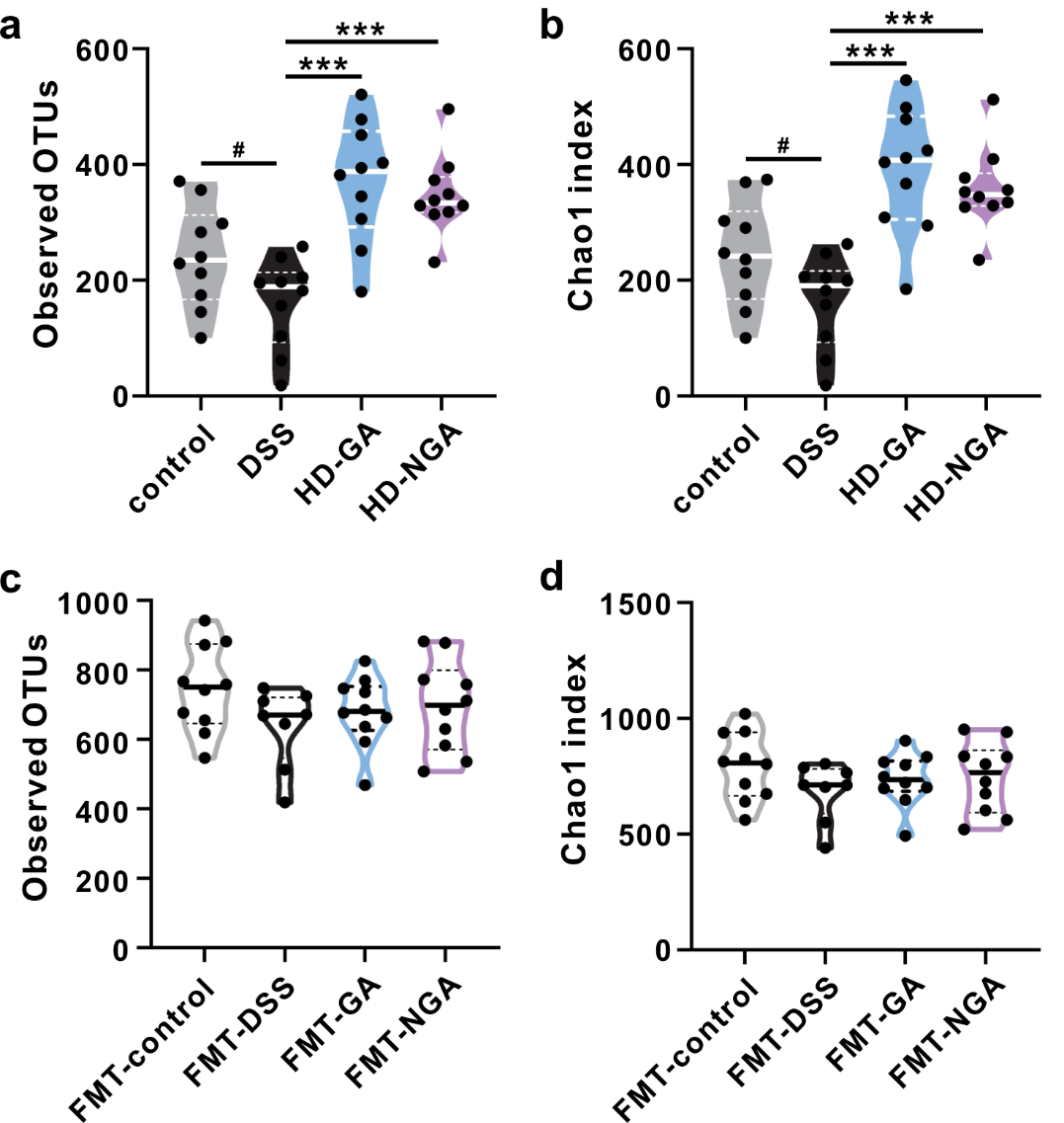


**Fig. S6** α-diversity of gut microbiota in compounds supplementation and FMT experiments. Observed OTUs index in **(a)** compounds supplementation and **(c)** FMT experiments. Chao1 index in **(b)** compounds supplementation and **(d)** FMT experiment. Data was represented as mean ± SD. # *p*<0.05, *** *p*<0.001.


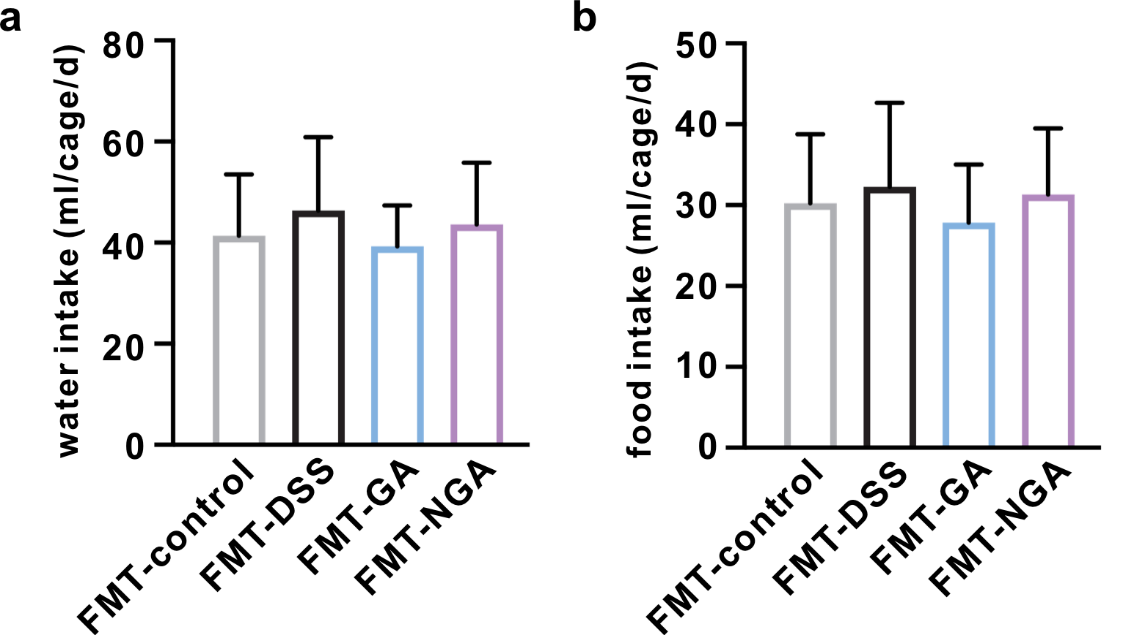


**Fig. S7** Water intake and food intake of each group in the FMT experiment. Data was represented as mean ± SD.


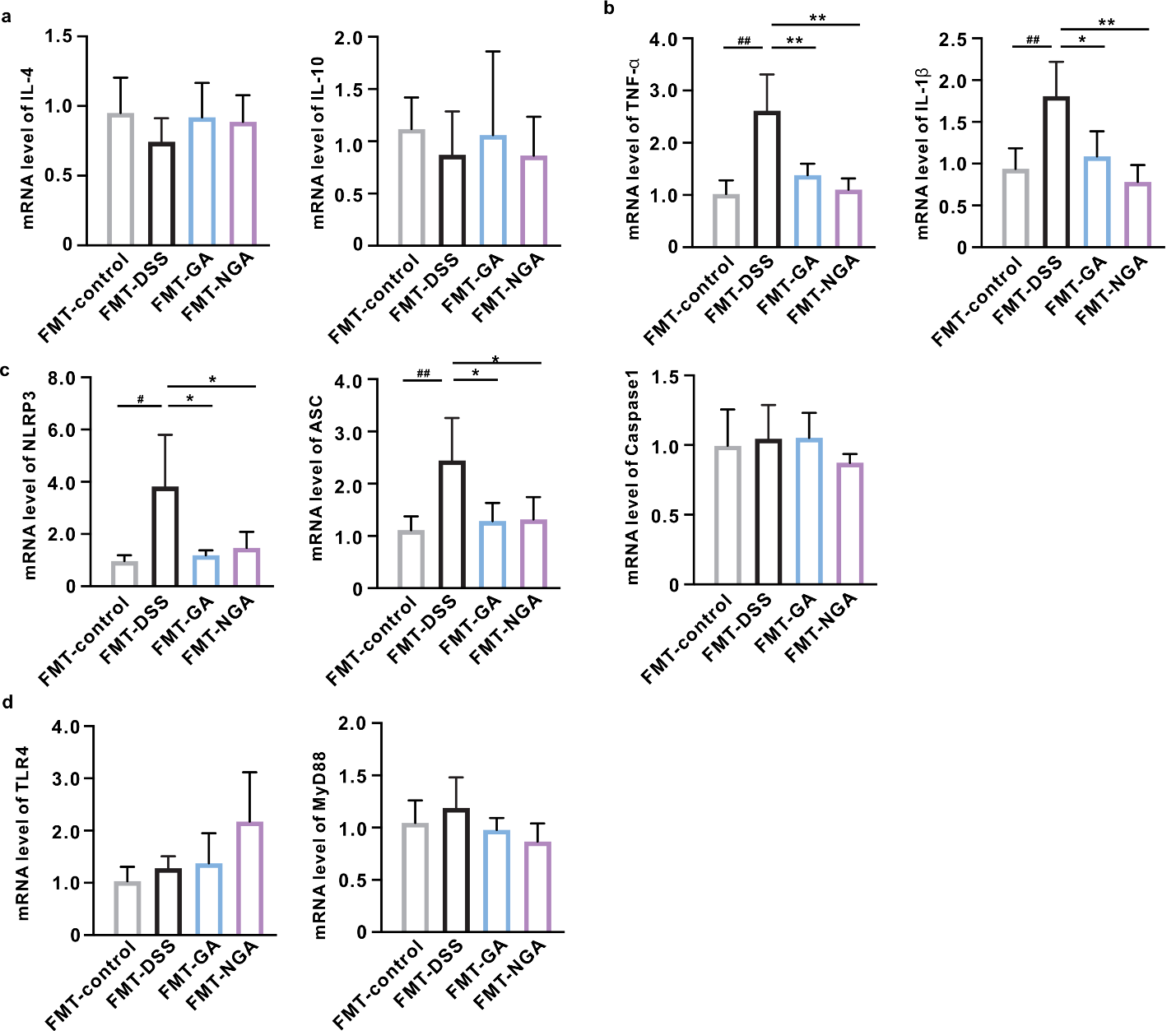


**Fig. S8** mRNA levels of key genes involved in signaling pathways in FMT experiment. **(a)** mRNA levels of IL-4 and IL-10 and **(b)** TNF-α and IL-1β. **(c)** mRNA expression level of NLRP3, ASC and Caspase1. **(d)** mRNA expression level of TLR4 and MyD88. Data was represented as mean ± SD. *, # *p*<0.05, **, ## *p*<0.01.


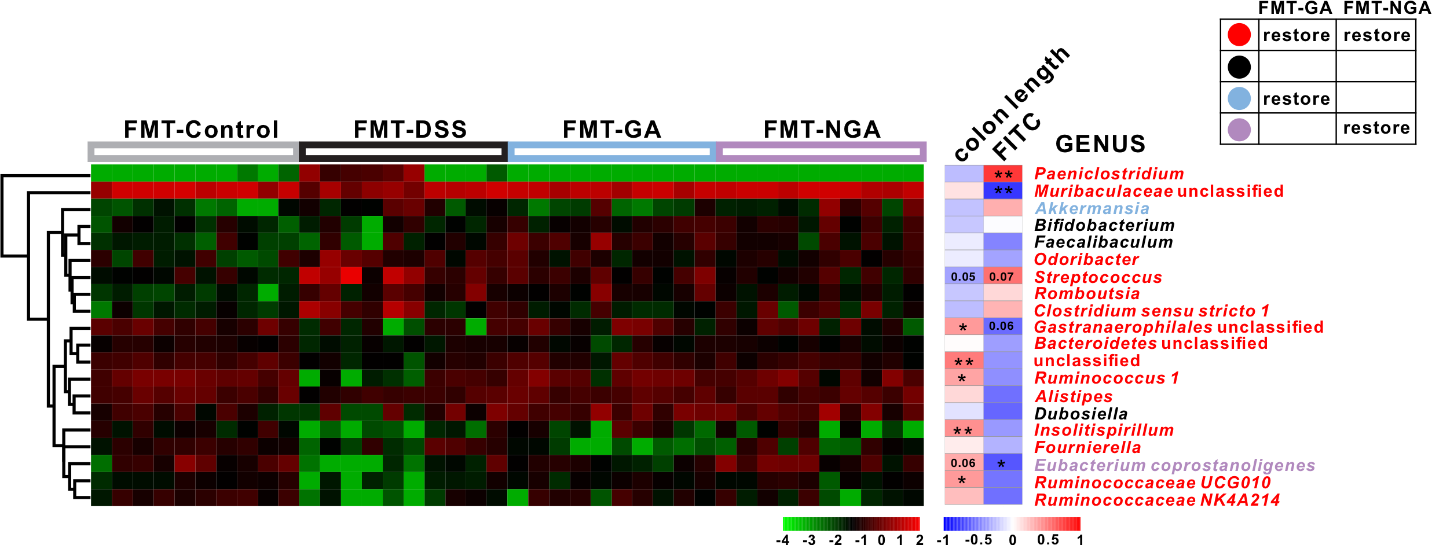


**Fig. S9** Heatmap of 20 key genera responding to FMT treatments and correlation study between gut microbiota and colitis-related indexes. * *p*<0.05, ** *p*<0.01.
